# Zinc oxide nanoparticles effectively regulate autophagic cell death by activating autophagosome formation and interfering with their maturation

**DOI:** 10.1186/s12989-020-00379-7

**Published:** 2020-09-18

**Authors:** Zixuan Liu, Xuying Lv, Lei Xu, Xuting Liu, Xiangyu Zhu, Erqun Song, Yang Song

**Affiliations:** Key Laboratory of Luminescence Analysis and Molecular Sensing (Southwest University), Ministry of Education, College of Pharmaceutical Sciences, Southwest University, Beibei, Chongqing, 400715 People’s Republic of China

**Keywords:** ZnO nanoparticles, Autophagic cell death, Autophagic flux, JNK, Autophagosomal-lysosomal fusion

## Abstract

**Background:**

With the development of zinc oxide nanoparticles (ZnO NPs) in the field of nanotechnology, their toxicological effects are attracting increasing attention, and the mechanisms for ZnO NPs neurotoxicity remain obscure. In an attempt to address concerns regarding neurotoxicity of ZnO NPs, we explored the relationship between free zinc ions, reactive oxygen species (ROS) and neurotoxic mechanisms in ZnO NPs-exposed PC12 cells.

**Result:**

This study demonstrated the requirement of free zinc ions shed by ZnO NPs to over generation of intracellular ROS. Next, we identified autophagic cell death was the major mode of cell death induced by ZnO NPs, and autophagosome accumulation resulted from not only induction of autophagy, but also blockade of autophagy flux. We concluded that autophagic cell death, resulting from zinc ions-ROS-c-Jun N-terminal kinase (JNK)-autophagy positive feedback loop and blockade of autophagosomal-lysosomal fusion, played a major role in the neurotoxicity of ZnO NPs.

**Conclusion:**

Our study contributes to a better understanding of the neurotoxicity of ZnO NPs and might be useful for designing and developing new biosafety nanoparticles in the future.

## Background

Nanoparticles (NPs) have been widely used in recent years, and the global market for nanotechnology products was estimated to reach 64.2 billion in 2019. Zinc oxide nanoparticles (ZnO NPs) are one of the most abundantly used metal oxide NPs, and global annual output of ZnO NPs was estimated at 0.1–1.2 million tons [1]. Due to the optical properties that combine broad-spectrum UV filtering with transparency to visible light, ZnO NPs are widely formulated into sunscreens [2]. ZnO NPs are also used for dental fillers [3] or food packaging materials to prevent bacterial contamination, because of their excellent antibacterial performance [4]. In addition, ZnO NPs have been utilized in semiconductors, coatings, and nanopiezotronics [5].

Although, the toxicities of ZnO NPs in mammalian organs, e.g. stomach, kidney, liver, spleen, and pancreas, have been widely studied [6], their potential hazardous effects on the central nervous system (CNS) are still limited. ZnO NPs may transfer to the CNS through blood brain barrier (BBB) [7], placental barrier [8], olfactory bulb or taste nerve pathways [9], leading to various neurotoxic effects. There are three inducers engaged in toxicity activities of ZnO NPs: the release of zinc ions from ZnO NPs, the production of reactive oxygen species (ROS) and mechanical damages caused by direct contact between the particles itself and the cells [10]. However, the regulatory mechanisms of ZnO NPs in various kinds of cells are different, for instance, inflammatory response [11], endoplasmic reticulum (ER) stress [12], necrosis [13], apoptosis [14], and autophagy [15] seem to be involved in the toxicity of ZnO NPs. Thus, understanding the possible mechanisms in determining the neurotoxicity of ZnO NPs is crucial for the safe use of ZnO-based nanomaterials.

Autophagy, specifically macroautophagy, is a regulatory process by which damaged organelles and proteins are delivered for lysosomal degradation [16]. Autophagy is a lysosomal-dependent degradation and recycling pathway. The conversion of microtubule-associated protein 1 light chain 3B (LC3B)-I to LC3B-II is often used as a biomarker for autophagy [16]. The dynamic process of autophagosome induction, fusion of autophagosomes with lysosomes and completion of lysosomal degradation is known as autophagic flux [17]. ZnO NPs are taken up into the cells by the ubiquitous endocytosis pathway and internalized into endosomal compartments. Endosomes tend to fuse into lysosomes, where ZnO NPs may be degraded to zinc ions due to the local acid environment. And then zinc ions are mobilized into the cytoplasm, resulting in zinc ions dyshomeostasis [10]. Thus, lysosomes associate the cellular response of ZnO NPs with autophagy. To date, autophagy is widely accepted as an important cellular response to NPs [18].

The role of autophagy in ZnO NPs cytotoxicity is controversial. Some studies consider that autophagy play a protective role in the presence of ZnO NPs [19, 20], while others suggest that autophagy inversely enhances ZnO NPs-induced death [21, 22]. This disparity might be due to the differences in ZnO NPs doses, characteristics, or tested cell types. However, the underlying mechanisms of autophagy during ZnO NPs-triggered neurotoxicity are poorly defined. Thus, the aim of this work was to elucidate the toxic effects and intrinsic mechanisms of ZnO NPs in vitro.

PC12 cell line, one of the widely used in vitro experimental models, has neuronal characteristics, such as expressing dopaminergic markers, and is capable of undergoing a variety of regulated cell death, such as apoptosis [23], necrosis [24], autophagy [25] and ferroptosis [26]. Thus, PC12 cells are often used to study the pathogenesis of many neurological diseases depending on the external chemicals [26, 27]. In the current study, we used culture PC12 cells and found the neurotoxicity of ZnO NPs developed in an ions-dependent manner, causing oxidative stress. And we demonstrated autophagic cell death was the major mode of cell death in ZnO NPs-treated PC12 cells. We further clarified that ZnO NPs-induced accumulation of autophagosomes resulted from not only induction of autophagy by c-Jun N-terminal kinase (JNK) activation, but also blockade of autophagic flux by decreasing autophagosomal-lysosomal fusion. In addition, ZnO NPs could be transported into lysosomes through the autophagy pathway, which further aggravated the oxidative toxicity induced by ions release and formed a positive feedback regulation.

## Materials and methods

### Nanoparticles

ZnO NPs (> 99.9% purity) were purchased from Macklin (Beijing, China). ZnO NPs were suspended in culture medium and ultrasonic for 15 min. Then the solution was diluted with a medium to the indicated concentrations and vigorously swirled for 30 s before cell exposure to avoid aggregation of nanoparticles.

### Reagents

N-(6-methoxy-8-quinolyl)-*p*-toluenesulfonamide (TSQ) and Fluor™Zn-520 were purchased from AAT Bioquest (California, USA). LysoSensor™ Green DND-189 and LysoTracker Red DND-99 were supplied by Yeasen Biotech (Shanghai, China). Propidium iodide (PI) and Hoechst 33258 were supplied by Solarbio (Beijing, China). 2′, 7′-dichlorodihydrofluorescein diacetate (DCFH-DA), *N*-Acetyl cysteine (NAC), N,N,N′,N′-Tetrakis (2-pyridylmethyl) ethylenediamine (TPEN), monodansylcadaverine (MDC), Ferrostatin-1 (Fer-1), Liproxstatin-1 (Lip-1) and antibody to LC3B were obtained from Sigma-Aldrich (Missouri, USA). Chloroquine (CQ) and Z-VAD-FMK were purchased from MedChemExpress (New Jersey, USA). 3-Methyladenine (3-MA), Necrostatin 1 (Nec-1), Necrosulfonamide (NSA), SP600125, SB203580 and PD98059 were obtained from Selleckchem (Texas, USA). Antibodies to p38 mitogen activated protein kinase (p38), extracellular signal regulated kinase 1/2 (ERK1/2) and JNK were purchased from Wanleibio (Shenyang, China). Antibody to Ras-related protein Rab-7a (Rab7) was supplied by Biosynthesis Biotechnology (Beijing, China). Antibodies to p62, Beclin 1, B cell lymphoma 2 (Bcl-2) and glyceraldehyde-3-phosphate dehydrogenase (GAPDH) were obtained from Proteintech (Wuhan, China). Antibodies to p-ERK (Tyr204), p-p38 (Thr180/Tyr182), p-JNK (Thr183/Tyr185), p-Bcl-2 (Ser70), lysosomal-associated membrane protein type 1 (LAMP-1) and LAMP-2 were obtained from Santa Cruz Biotechnology (Santa Cruz, CA). Cytoplasmic and Mitochondrial Protein Extraction Kit was obtained from Sangon Biotech (Shanghai, China). Dulbecco’s modified Eagle’s medium (DMEM) and trypsin were supplied by Keygen Biotech (Nanjing, China). All other chemicals used were of the highest commercial grade.

### Transmission Electron Microscopy (TEM)

For ZnO NPs TEM detection, ZnO NPs were suspended in deionized water at a concentration of 3 mg/mL and sonicated for 30 min. Then the suspension was diluted with deionized water to a concentration of 30 μg/mL and swirled vigorously for 5 min before TEM detection.

For TEM detection of PC12 cells morphology, the morphology and microstructure of PC12 cells were observed by TEM as described previously [28]. Briefly, after ZnO NPs exposure, cells were collected by centrifugation (800 g) and fixed in 4% glutaraldehyde, post-fixed with 1% OsO_4_ and then embedded in Epon. Finally, Hitachi-7500 TEM instrument (Hitachi, Japan) was used to examine the ultrathin sections stained with uranyl acetate/lead citrate.

### Zinc ions release assay

After exposed to ZnO NPs for 24 h, 5 × 10^7^ cells were collected by centrifugation at 600 g for 5 min and washed by re-suspending cells with PBS. For dissociated zinc ions concentrations measurement, the cells were lysed with liquid nitrogen and centrifuged (15, 000 g, 30 min) to remove the NPs. Then, the supernatant was used to measure the concentration of dissociated zinc ions and proteins. For the total intercellular zinc concentrations, the cells were divided into two groups. One group was used to determine protein content, and the other was used to quantify the zinc element after digestion with HNO_3_ and H_2_O_2_. The zinc concentration was determined using atomic absorption spectroscopy (AAS) in the graphite furnace mode (TAS-990, Persee, China). Each experiment was performed in triplicate.

### Cell culture and exposure

Rat pheochromocytoma PC12 cell line (American Type Culture Collection, USA) were grown in DMEM supplemented with 10% heat-inactivated newborn bovine serum (EVERY GREEN, China), 100 U/mL penicillin, and 100 μg/mL streptomycin. It was maintained at 37 °C in a humidified incubator containing 5% CO_2_. For differentiation, PC12 cells were treated with 50 ng/mL never growth factor (NGF, Sigma, USA) for 8 days. Then cells were collected and used in experiments. In rescue experiments, the inhibitors of signaling pathways were pretreated with cells for 1 h, then 15 μg/mL ZnO NPs was added to cell culture medium for the indicated times.

### Determination of cell viability

Cell viability was determined by the Cell Counting Kit-8 (CCK-8) Assay (Bimake, USA). Briefly, cells were seeded in 96-well culture plates at a density of 5 × 10^3^/well for 24 h. For determination of the viability after exposure to 15 μg/mL ZnO NPs for the indicated times, detection reagent, made up of fresh medium containing 10% kit reagent, was added to cells and followed by 1–4 h incubation. The absorbance was measured using the ELx800 microplate reader (BioTek Instruments, USA) at a wavelength of 450 nm.

### Confocal microscopy

For detecting the subcellular location of zinc ions after exposed to ZnO NPs for 12 h, the cells were rinsed three times with PBS followed by staining with 30 μM Zn^2+^-specific fluorescent dye TSQ for 20 min. Then the lysosomes were stained by 50 nM LysoTracker Red DND-99 for 30 min followed by rinsing three times with PBS. Finally, the pictures were taken by the confocal microscope (Nikon, Japan) with an excitation wavelength of 405 nm for zinc ions and 561 nm for lysosomes.

For detecting the fluorescent protein-light chain 3 (GFP-LC3) puncta induced by ZnO NPs, the cells were transfected with GFP-LC3 plasmid, using RNAi-Mate (GenePharma, China) according to the manufacturer’s instructions. Briefly, GFP-LC3 plasmid and RNAi-Mate were mixed together, and left at room temperature for 20 min to form the complexes, which was then added into the cells for 24 h before further exposure. The pictures of PC12 cells were taken by the confocal microscope (Nikon, Japan) with an excitation wavelength of 488 nm for GFP-LC3 puncta.

For detecting the lysosomal characters after exposed to ZnO NPs for 12 h, the cells were stained by 50 nM LysoTracker Red DND-99 for 30 min, followed by staining with 1 mg/mL Hoechst 33258 for 20 min in the dark. After rinsing three times with PBS, the pictures were taken by the confocal microscope (Nikon, Japan) with an excitation wavelength of 405 nm for nucleus and 561 nm for lysosomes.

### Flow cytometry analysis

For evaluating the uptake of ZnO NPs by PC12 cells, side scatter (SSC) intensity, reflecting the relative complexity of cells, was detected. Briefly, after 2 h of ZnO NPs exposure, PC12 cells were washed twice with PBS and collected with trypsin. Then cells were resuspended with fresh DMEM and the SSC was immediately detected.

For zinc ions detection, a zinc ions-specific indicator Fluor™Zn-520 was used. After ZnO NPs exposure, cells were collected to incubate with Fluor™Zn-520 at a final concentration of 1.5 μM, followed by 30 min incubation at 37 °C in the dark. Cells were then washed twice with PBS to remove excess fluorescent probes. After cells were resuspended, fluorescence intensity was detected by flow cytometry.

For assessing the toxicity of ZnO NPs, PI staining were employed to detect the cells with damaged cell membranes. After ZnO NPs exposure, cells were harvested with trypsin and washed twice with PBS. Then, the cell pellet was resuspended with fresh DMEM medium containing 3 μM PI staining solution. After incubation for 15 min at room temperature, the stained cells were directly analyzed by flow cytometry.

For measuring ROS, an oxidation-sensitive fluorescence probe DCFH-DA was used. Briefly, cells were collected and washed twice with PBS. Then cells were incubated with 10 μM DCFH-DA at 37 °C for 30 min. After cells were resuspended, fluorescence intensity was detected by flow cytometry.

For evaluating the level of autophagosome accumulation, MDC staining, a tracer of autophagic vesicles, was used. Briefly, cells were collected and incubated with 50 μM MDC for 30 min in the dark. After staining, the cells were washed three times with PBS and immediately determined by flow cytometry.

For lysosome damage detection, LysoTracker Red DND-99 for lysosomes and LysoSensor™ Green DND-189 for lysosomal pH were used. After ZnO NPs exposure, cells were stained by 50 nM LysoTracker Red DND-99 or 2 μM LysoSensor™ Green DND-189 for 30 min, respectively. Then cells were resuspended and processed for flow cytometry.

In all the experiments, fluorescence was determined with a BD FACS Melody™ flow cytometry and the results were analyzed using the FlowJo software. FACS histogram showing the distribution of fluorescence intensity or histogram showing the average fluorescence intensity was presented.

### Protein extraction and Western blotting analysis

PC12 cells were collected at an indicated time after exposure to ZnO NPs. Then, proteins were prepared with RIPA lysis buffer [150 mM NaCl, 50 mM Tris-HCl (pH 7.4), 0.1% SDS, 1% NP-40, 0.5% sodium deoxycholate] containing protease inhibitors and phosphatase inhibitors. The total protein concentration was determined with a BCA assay kit (Keygen Biotech, China). Proteins were separated by sodium dodecyl sulfate polyacrylamide gel electrophoresis (SDS-PAGE) and then transferred onto nitrocellulose membranes. Prior to incubation with primary antibodies at 4 °C overnight, the membranes were blocked with skim milk for 1 h at the room temperature. Finally, the membranes were incubated with horseradish peroxidase-conjugated secondary antibodies at room temperature for additional 1 h. Detection was performed using the BeyoECL Star (Beyotime Biotechnology, China). Densitometry was carried out using ImageJ software from at least three separate experiments and GAPDH was used as a housekeeping gene in the experiments.

### Immunoprecipitation (IP)

PC12 cells were treated with ZnO NPs for 6 h. Then, cells were lysed, centrifuged, and the supernatants were collected. After the concentration of the lysates was detected, the supernatant was divided into two parts, one was used as the whole cell extracts (Input), and the other was used for IP experiment. IP experiments were performed with protein A/G magnetic beads (Bimake, USA) according to the manufacturer’s instructions. Briefly, the beads/Beclin 1 antibody complex (10 μg antibody and 50 μL protein A/G magnetic beads) was prepared, and then the cell lysates and beads were incubated at 4 °C for 12 h. After washing with lysis buffer, the protein complexes were boiled in 1 × SDS loading buffer and subjected to Western blotting as previously described.

### Statistical analysis

All data generated by at least three independent experiments, were presented as mean ± standard deviations (SD). The statistical significance of the differences was analyzed in GraphPad Prism 7.0 software by one-way ANOVA followed by Tukey’s multiple comparisons test, and *p* values less than 0.05 was considered statistically significant.

## Results and discussion

### The uptake and ions-shedding ability of ZnO NPs in PC12 cells

The morphology and characteristics of ZnO NPs used in this study were measured in Figure S1A, B and summarized in Table S1. The results demonstrated that their shape was irregular. The TEM size (length 180 nm, diameter 95 nm) was smaller than the hydrodynamic size, and the hydrodynamic diameter was ≈262 nm in water and ≈585 nm in cell culture medium, indicating the particles were slightly aggregated in cell culture medium. Then, we examined the zinc ions release process of ZnO NPs through detecting the change of free zinc ions levels over time. Zinc ions concentration was measured using AAS. As shown in Figure S1C, the dissolution of ZnO NPs in complete DMEM medium was higher than in water, suggesting biologically relevant buffering system impacted the dynamics of ZnO NPs dissolution.

**Fig. 1:**
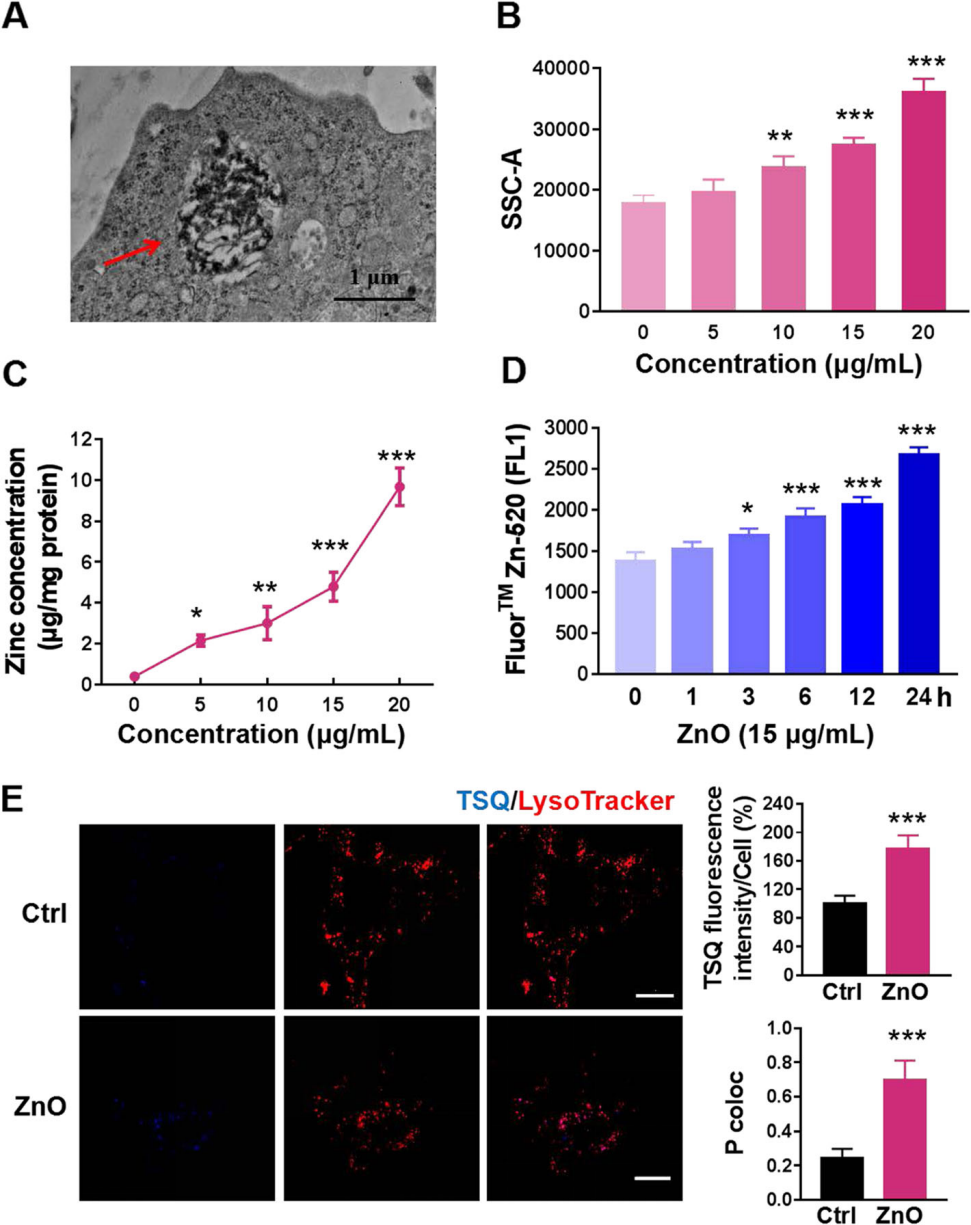
The uptake of ZnO NPs and the release of zinc ions from ZnO NPs. **a** TEM image of ZnO NPs internalized in PC12 cells. PC12 cells were treated with 15 μg/mL ZnO NPs for 6 h. Red arrows indicated that ZnO NPs were wrapped into cells. Scale bar, 1 μm. **b** Exposure to different doses (5, 10, 15 and 20 μg/mL) of ZnO NPs for 2 h showed a particle-specific internalization. The mean SSC-A was analyzed by flow cytometry to represent the uptake of ZnO NPs. **c** AAS quantification of the uptake of ZnO NPs. PC12 cells were exposed to various concentrations of ZnO NPs for 24 h. Total cellular zinc content was detected as described in “Materials and Methods” and expressed as μg zinc element per mg of cellular proteins. **d** Fluor™ Zn-520 probe for measurement of intracellular free Zn^2+^ concentrations. After incubation of PC12 cells with 15 μg/mL ZnO NPs for the indicated times, the cells were loaded with Fluor™ Zn-520, then the fluorescence intensity was measured by flow cytometry. **e** Distribution of labile zinc ions in PC12 cells. Cells were treated with 15 μg/mL ZnO NPs for 12 h. Intracellular co-localization of zinc ions (TSQ probe, blue) with lysosomes (Lyso tracker, red) was imaged using the confocal microscopy and determined by calculation of Pearson’s correlation coefficient (P coloc) of 27 cells. Scale bar, 20 μm. Data from at least three independent experiments were expressed as the means ± SD. *p < 0.05, **p < 0.01, ***p < 0.001 compared with the untreated control

In order to investigate the neurotoxicity of ZnO NPs, we first detected the ability of PC12 cells to internalize ZnO NPs by means of TEM and by analyzing SSC shift using flow cytometry. TEM analysis confirmed that ZnO NPs were accumulated in cytoplasmic region and formed a phagophore-like structure (Fig. 1a). SSC intensity, which represents the granularity of cells, showed a significantly increased uptake of ZnO NPs in a dose-dependent manner at 2 h (Fig. 1b). Quantitative analysis by AAS measured the total zinc content of the cells, including particles as well as zinc ions, and showed that total zinc element *per* mg of cellular proteins increased in a dose-dependent manner after exposure to ZnO NPs (Fig. 1c). These data indicated that ZnO NPs were absorbed by PC12 cells. It has been reported that the toxic effect of ZnO NPs is caused by their dissociation and dissolution of zinc ions, which disrupt cellular zinc homeostasis and ultimately lead to cell death [29, 30]. Hence, we examined the intracellular free zinc ions shed by ZnO NPs using Fluor™Zn-520, a specific fluorescent indicator for zinc ions. Intracellular zinc ions signal values continued to increase over time in PC12 cells (Fig. 1d). In addition, there was a significant overlap between zinc ions and lysosomes, as the Pearson correlation coefficient values was 0.7002 (Fig. 1e). Mechanically, ZnO NPs accumulate on cell membrane and traverse through the membrane by endocytosis, then intracellular traffic to the acidic lysosomes for the release of zinc ions from ZnO NPs.

### ZnO NPs-induced oxidative death is iron dependent

**Fig. 2:**
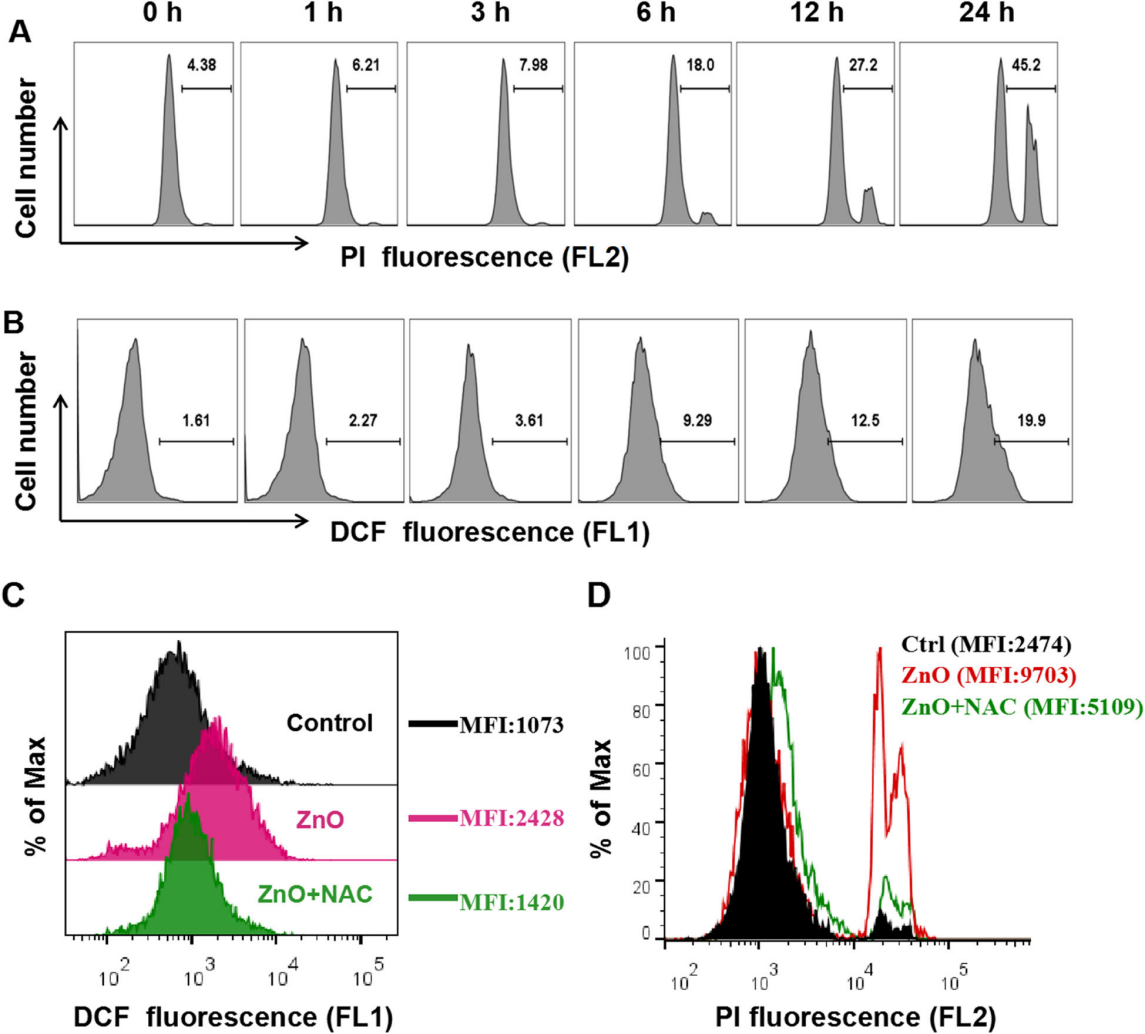
The role of ROS in ZnO NPs-induced cytotoxicity. **a** PI staining to show the cytotoxicity of ZnO NPs in PC12 cells. These cells were exposed to 15 μg/mL ZnO NPs for the indicated times, and the percentage of dead cells was measured by PI staining. Flow cytometry results were displayed in histogram plot. **b** ROS levels after ZnO NPs exposure were detected using the ROS indicator DCF. Effect of NAC on ZnO NPs-induced ROS level by DCFH-DA staining (**c**) and cytotoxicity by PI staining (**d**). Cells were pretreated with 2 mM NAC for 1 h, followed by exposure to 15 μg/mL ZnO NPs for 6 h or 12 h, respectively. MFI: mean fluorescence intensity. Data were representative of at least three independent experiments

For toxicity testing, PI staining was employed to detect the damaged cells. As shown in Fig. 2a, with the increase of incubation time of ZnO NPs, the percentage of PI positive cells was increased, which was consistent with the increase of zinc ions (Fig. 1d). Xia et al. reported that the production of ROS is a major toxicological paradigm for ambient and engineered NPs, including ZnO NPs [29, 31]. In our experiment, the increasing trend of ROS over time was consistent with the degree of cell damage analyzed by PI staining during ZnO NPs exposure (Fig. 2b). In addition, the pretreatment with NAC, which cleared out excessive ROS after ZnO NPs exposure (Fig. 2c), significantly ameliorated the cell damage caused by ZnO NPs (Fig. 2d). These results indicate that ROS is the key mediator of ZnO NPs-induced cytotoxicity.

**Fig. 3:**
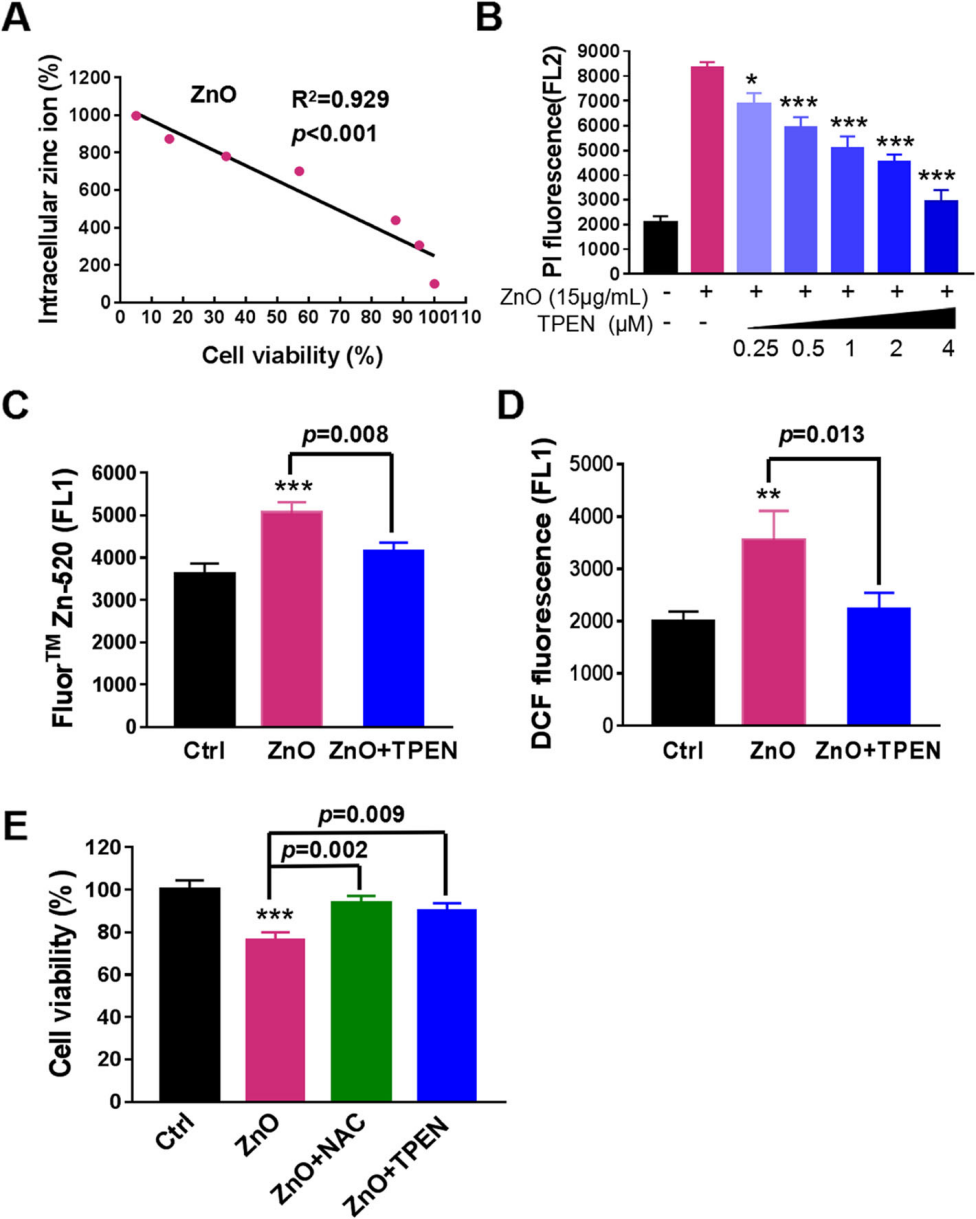
The role of zinc ions in ZnO NPs-induced oxidative stress. **a** Intercorrelation of cell viability and intracellular free zinc ions levels after exposure to ZnO NPs for 24 h. **b** Effect of TPEN on the cytotoxicity of ZnO NPs by PI staining. Cells were pretreated with the indicated doses of TPEN for 30 min, and then incubated with 15 μg/mL ZnONPs for 6 h. Effect of TPEN on intracellular zinc ions by zinc ions indicator Fluor™ Zn-520, AM staining **(c)** and ROS level by DCFH-DA staining **(d)** in ZnO NPs-treated PC12 cells. Cells were pretreated with 4 μM TPEN for 30 min, followed by exposure to 15 μg/mL ZnO NPs for 6 h. **e** Effects of NAC and TPEN on the cytotoxicity of ZnO NPs by CCK8 assay. Cells were pretreated with 2 mM NAC or 4 μM TPEN for 30 min, and then cell viability was determined at 12 h after 15 μg/mL ZnO NPs exposure. Data from at least three independent experiments were expressed as the means ± SD. *p < 0.05, **p < 0.01, ***p < 0.001 compared with the untreated control

We next explored the relationship between free zinc ions and cell death in PC12 cells. A strong correlation between ZnO NPs-induced cell viability and available intracellular zinc ions levels was observed (R^2^ = 0.9288) across all exposure concentrations of ZnO NPs (Fig. 3a). The original cell viability statistics and quantitative analysis of intracellular dissolution of ZnO NPs were shown in Figure S2A and B. A similar correlation was also observed in ZnCl_2_-evoked PC12 cells (Figure S2C). Although the toxicity of ZnCl_2_ was slightly weaker than that of the same molar concentration of ZnO NPs, presumably due to the higher level of available zinc ions in ZnO NPs-treated cells. Moreover, TPEN, a specific zinc ions-chelating agent, could reduce cell damage in a dose-response effect (Fig. 3b), implicating a requirement for zinc ions rather than NPs themselves to precede neurotoxicity. The elevated cytosolic zinc ions would sequestered by mitochondria [32], resulting in mitochondrial dysfunction and the generation of ROS. To further explore the potential mechanisms of zinc ions-mediated neurotoxicity of ZnO NPs, we then investigated the relationship between free zinc ions and ROS in PC12 cells. TPEN treatment significantly reduced the concentration of zinc ions in cells (Fig. 3c), while alleviating the excessive ROS produced by ZnO NPs (Fig. 3d), indicating that elevated levels of ROS exposed to ZnO NPs were correlated to intracellular zinc ions. Furthermore, cell viability data revealed TPEN, similar to the effect of NAC, blocked the neurotoxicity of ZnO NPs (Fig. 3e). Compared with the results of Fig. 2d, the failure of NAC to completely block the uptake of PI induced by ZnO NPs may be due to the damage of the material itself to cell membrane, leading to PI infiltration into the cells. All considered, the data demonstrate that the intrinsic reason for the toxicity of ZnO NPs to PC12 cells is over generation of ROS induced by cell uptake of ZnO NPs, intracellular dissolution and release of zinc ions.

### ZnO NPs induce autophagosome accumulation, leading to autophagic cell death

**Fig. 4:**
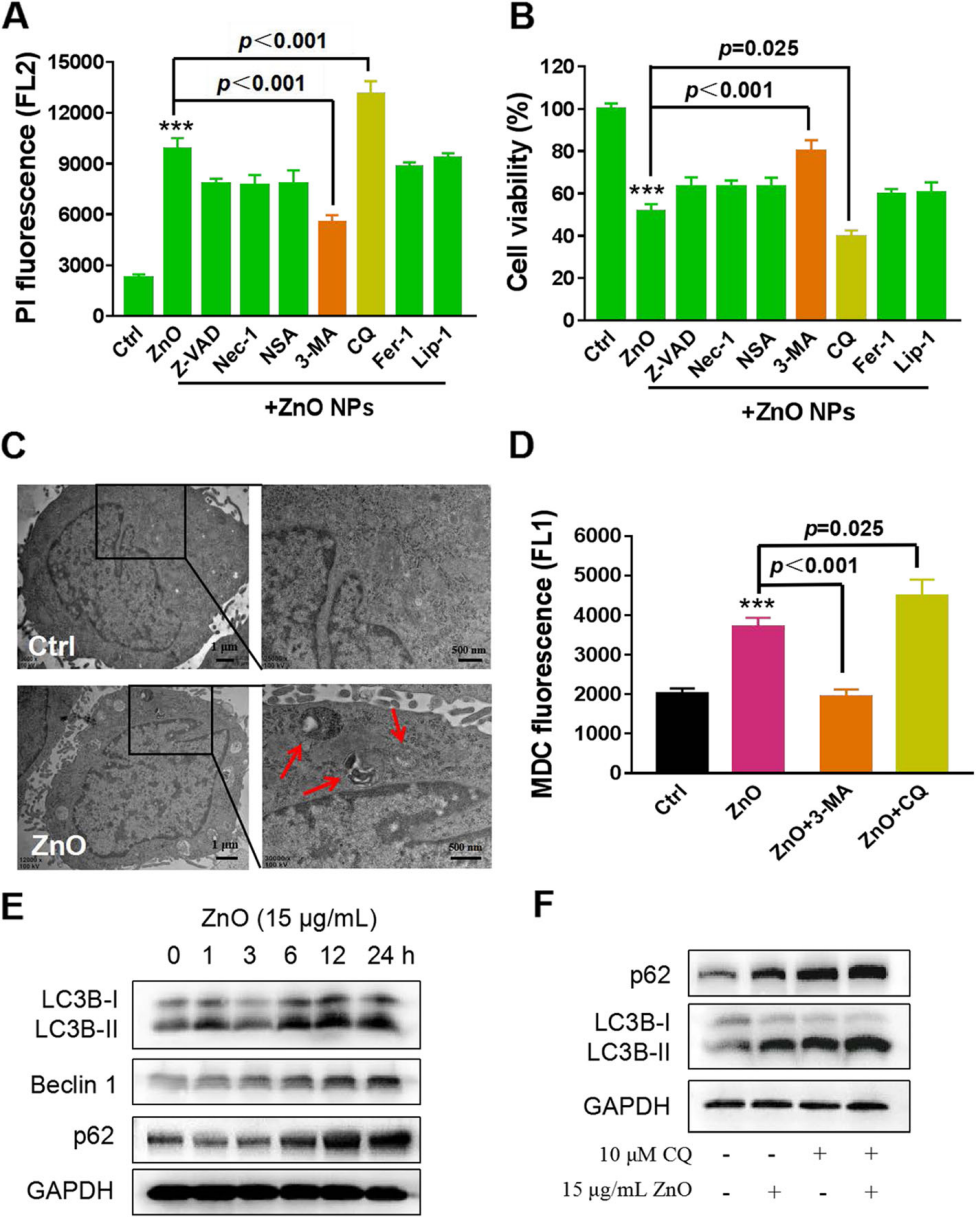
Abnormal accumulation of autophagosomes in ZnO NPs-treated PC12 cells. Effects of well-known cell death inhibitors on the cytotoxicity of ZnO NPs by PI staining **(a)** and by CCK8 assay **(b)**. PC12 cells were treated with 15 μg/mL ZnO NPs in the presence or absence of various cell death inhibitors, Z-VAD (Z-VAD-fmk, caspase inhibitor, 20 μM), Necrostatin 1 (Nec-1, RIPK1 inhibitor, 20 μM), Necrosulfonamide (NSA, MLKL inhibitor, 1 μM), 3-Methyladenine (3-MA, autophagosome formation inhibitor, 2 mM), Chloroquine (CQ, inhibitor of mature autophagosomes fusion with lysosomes, 10 μM), Ferrostatin-1 (Fer-1, lipid peroxidation inhibitor, 1 μM), Liproxstatin-1 (Lip-1, another lipid peroxidation inhibitor, 500 nM). **c** Representative TEM images of autophagosomes. PC12 cells were treated with 15 μg/mL ZnO NPs. Red arrows indicated autophagosomes. **d** Flow cytometry analysis of autophagosomes by MDC staining in ZnO NPs-induced PC12 cells for 6 h with or without 3-MA and CQ pre-treatment for 1 h. Data from three independent experiments were expressed as the means ± SD. ***p < 0.001 compared with the untreated control. **e** Effects of ZnO NPs on the expression of autophagy-related proteins. LC3B, Beclin 1 and p62 expression were examined by Western blotting. **f** Western blotting analysis of LC3B and p62 expression levels in PC12 cells treated with 15 μg/mL ZnO NPs for 6 h in the presence or absence of CQ pre-treatment for 1 h. Images were representative of at least three independent experiments, and the densitometry of those bolts was shown in Figure S8A and B

ZnO NPs can induce different cell death patterns in different situations, such as apoptosis [33] or non-apoptotic cell death [15]. To further explore mechanisms involved in ZnO NPs-induced neurotoxicity in PC12 cells, cell death was assessed by PI staining (Fig. 4a) and CCK-8 assay (Fig. 4b) following exposure to ZnO NPs in the presence of antagonists to the known cell death signals. Only 3-MA, an inhibitor of autophagosome formation, significantly increased the number of viable cells reduced by ZnO NPs, although other cell death inhibitors, such as apoptosis, necrosis and ferroptosis, had slight effects on alleviating the cytotoxicity triggered by ZnO NPs. However, CQ, which destroy lysosomal function by increasing lysosomal pH, accelerated cell death in combination with ZnO NPs. These results showed that the inhibition of autophagosome formation by 3-MA decreased cell death induced by ZnO NPs, and inhibition of autophagosome degradation by CQ sensitized cells to ZnO NPs-induced cell death. Therefore, we hypothesized that the toxicity of ZnO NPs was mainly related to the accumulation of autophagosomes. TEM micrographs showed the amount of autophagosomes was increased markedly in cells upon ZnO NPs exposure (Fig. 4c). This finding was consistent with the MDC staining observed in Fig. 4d**.** MDC accumulates in mature autophagic vacuoles, such as autophagosomes but not in the early endosomes, and is widely used as a specific marker for autophagy [34]. MDC staining showed increased accumulation of autophagosomes in ZnO NPs-treated cells compared with the control cells. And CQ significantly enhanced ZnO NPs-induced MDC fluorescence intensity, while 3-MA inhibited this effect.

To investigate the mechanisms of autophagosome accumulation, we investigated the expression level of LC3B-II, the key protein of autophagosomes. Western blotting analysis results showed that the levels of LC3B-II kept increasing during the ZnO NPs-treatment period. Simultaneously, ZnO NPs exposure enforced the expression of autophagy-related proteins Beclin 1 in a time-dependent manner (Fig. 4e). However, p62/SQSTM1, a specific substrate degraded by autophagy-lysosome pathway and reflecting autophagosomes turnover [35], began inversely increased at 6 h upon autophagy activation in ZnO NPs-treated PC12 cells, suggesting a blockade in autophagic flux (Fig. 4e). And the addition of CQ (a well-established method for blocking autophagic flux) further confirmed this phenomenon. As shown in Fig. 4f, CQ + ZnO NPs increased p62 protein level to the same degree as CQ alone with no synergistic effect, indicating that ZnO NPs-induced p62 protein accumulation was the result of impairment of autophagic flux, but not the result of p62 gene transcription or translation regulation.

Autophagosome accumulation could be due to either autophagy induction or defect of lysosomal degradation [36]. We have demonstrated the autophagic flux was blocked, and next we detected the role of autophagy induction. As shown in Fig. 4f, the co-treatment of ZnO NPs with CQ had a higher level of LC3B-II than treatment with CQ alone, indicating that the elevation of LC3B-II level induced by ZnO NPs was derived from autophagy induction. Taken together, our data demonstrate that ZnO NPs-induced the accumulation of autophagosomes resulted from the initiation of autophagy induction and blockade of autophagic flux, leading to autophagic cell death, the major mode of cell death in PC12 cells.

### JNK activation contributes to ZnONPs-induced PC12 cell death

**Fig. 5:**
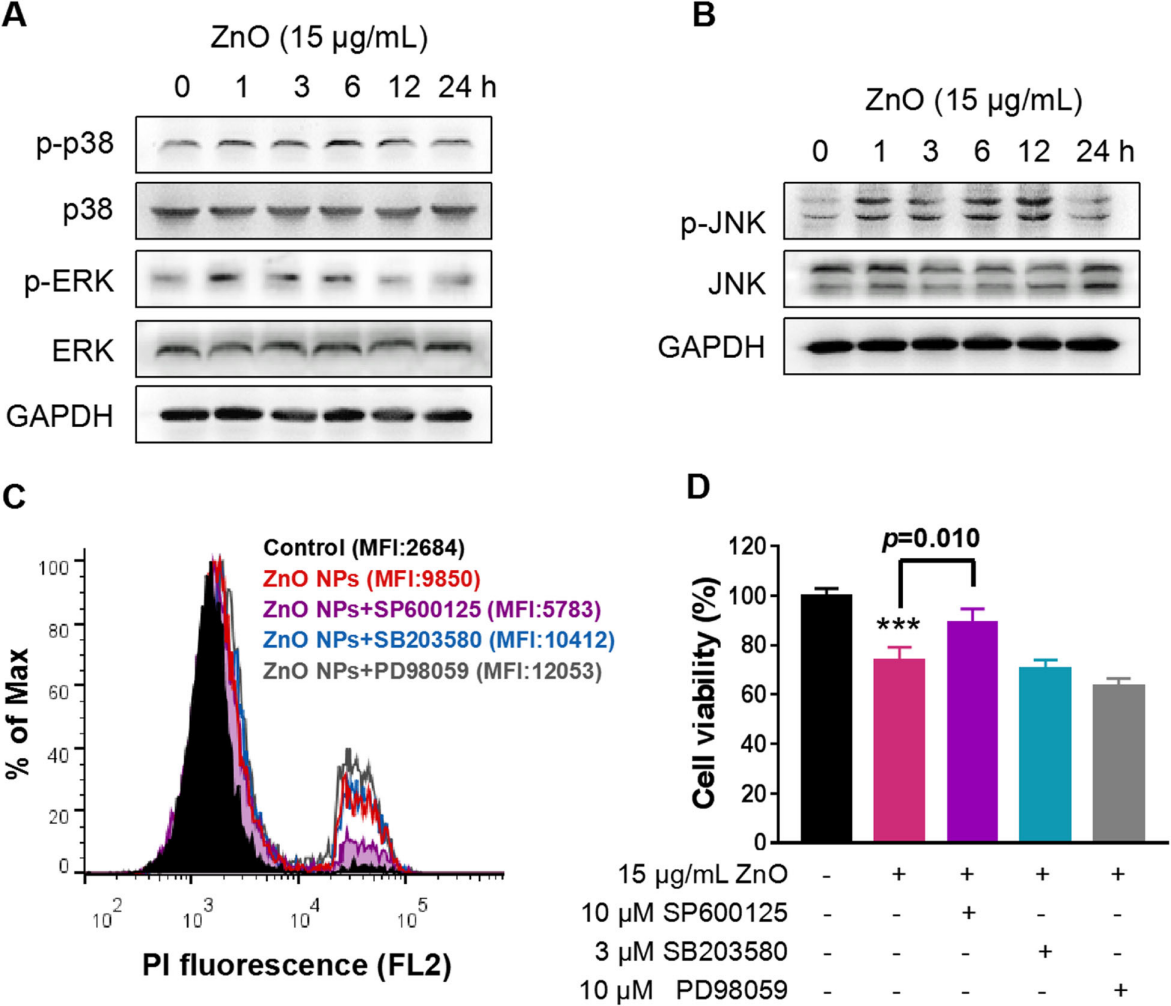
JNK activation contributes to ZnO NPs-induced PC12 cell death. Effects of ZnO NPs on p38, ERK **(a)**, and JNK **(b)** signaling pathways. Activation status of the p38, ERK, and JNK was analyzed in whole cell proteins by immunoblotting with antibodies to phosphorylated p38, ERK, JNK and total p38, ERK, JNK, respectively. The densitometry of those bolts from at least three independent experiments was shown in Figure S8C and D. Effect of JNK, p38 and ERK inhibitors on ZnO NPs-induced cell death as shown by PI staining **(c)** and CCK8 assay **(d)**. Cells were pretreated with 10 μM JNK inhibitor SP600125, 3 μM p38 inhibitor SB203580 and 10 μM ERK inhibitor PD98059 for 1 h, followed by exposure to 15 μg/mL ZnO NPs for 12 h. Data from at least three independent experiments were expressed as the means ± SD. ***p < 0.001 compared with the untreated control

Given the important role of autophagy in cytotoxicity induced by ZnO NPs, we next unraveled the molecular mechanisms of possible signaling pathways preceding autophagy. The known mechanisms regulating the autophagy process include the Akt/mammalian target of rapamycin (mTOR) [37, 38], AMP-activated protein kinase (AMPK) [39] and mitogen-activated protein kinases (MAPKs) pathways [40]. Our data manifested that ZnO NPs exposure initially reduced the phosphorylation of mTOR, but with the extension of time, the levels of mTOR phosphorylation recovered (Figure S3A), which was inconsistent with the continuous activation of LC3B (Fig. 4e). No alteration of AMPK phosphorylation induced by ZnO NPs was observed in the present study (Figure S3B). The above data suggested that there may be other pathways to regulate ZnO NPS-induced autophagy. MAPKs, which participate in the regulation of various life activities such as cell survival and death, have been demonstrated to be activated in response to a variety of external stimuli, including ZnO NPs [41–43]. In order to elucidate the involvement of MAPKs in ZnO NPs-induced autophagic cell death, we then assessed the activation status of three major classes in MAPK pathways following incubation with ZnO NPs in PC12 cells. ZnO NPs exposure had no effect on p38 MAPK phosphorylation. The phosphorylated forms of ERK (Fig. 5a), JNK (Fig. 5b) were rapidly increased within 1 h post exposure. Subsequently, the elevated level of phosphorylated form of c-Jun, a downstream protein of JNK, also demonstrated that ZnO NPs could induce JNK activation (Figure S4). In addition, to distinguish the effects of the specific MAPKs involved in ZnO NPs-induced cytotoxicity, we pretreated the cells with SP600125, SB203580, and PD98059, pharmacologic inhibitors of JNK, p38 and ERK, respectively. Pretreatment with SB203580 hardly altered ZnO NPs-mediated cytotoxicity. PD98059 aggravated the cytotoxicity of ZnO NPs, indicating that ERK mediated survival when ZnO NPs stimuli was applied. Only SP600125 selectively and effectively protected PC12 cells from ZnO NPs-evoked cytotoxicity as shown by PI staining (Fig. 4c) and CCK8 assay (Fig. 4d). These data suggest the activation of JNK is involved in ZnO NPs-induced PC12 cell death, but not the ERK and p38.

### JNK activation is required for autophagy induction following the stimulation of ZnO NPs

**Fig. 6:**
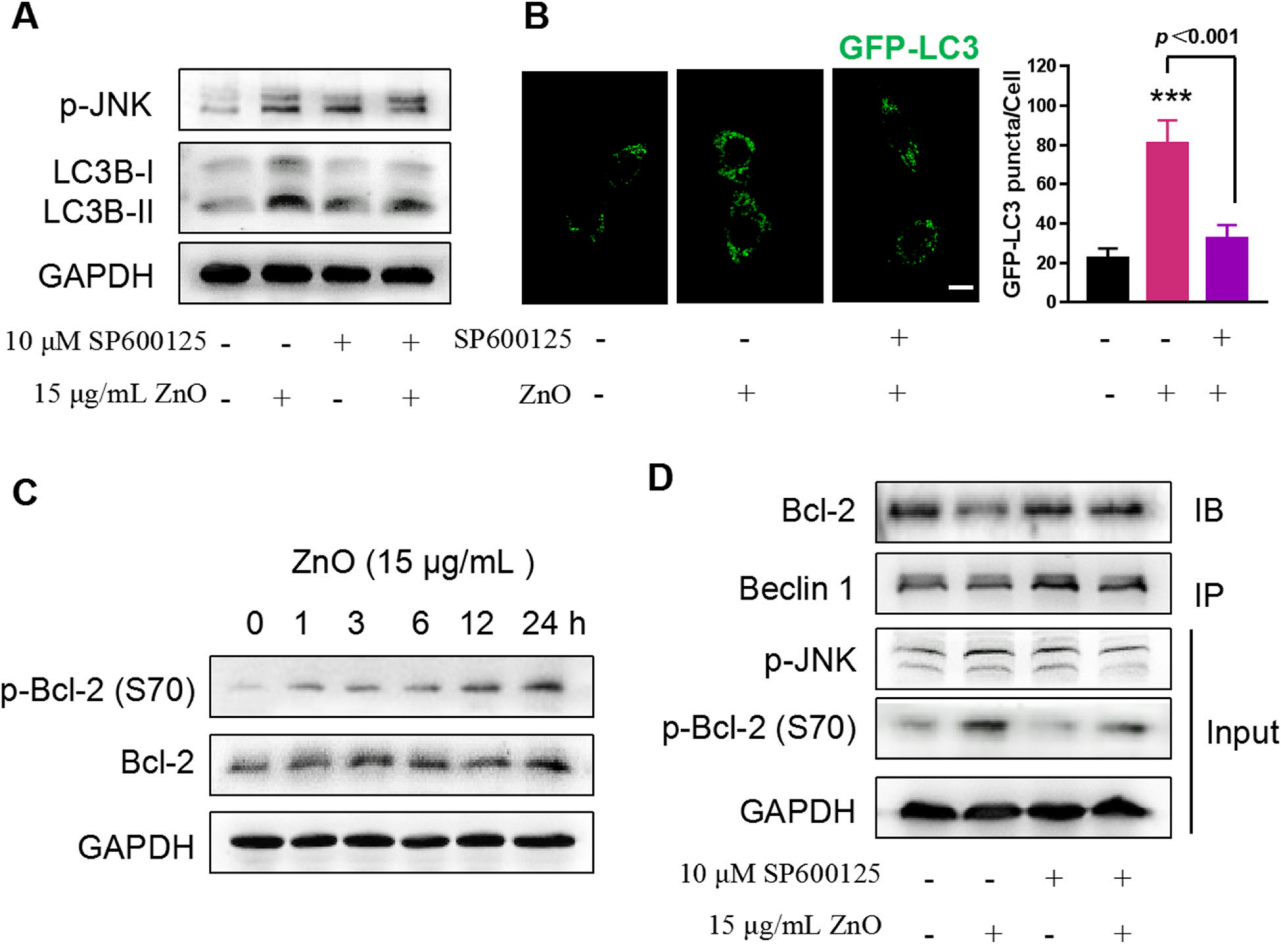
The autophagy regulation role of JNK signaling pathway in ZnO NPs-treated cells. PC12 cells were pretreated with 10 μM SP600125 for 1 h prior to 6 h exposure to 15 μg/mL ZnO NPs. **a** The inhibitory effects of SP600125 on the expression of p-JNK and LC3B were determined by Western blotting. **b** Representative images and statistical analysis of PC12 cells with GFP-LC3 puncta. Cells were transiently transfected with GFP-LC3 plasmid. The inhibitory effect of SP600125 on autophagosome accumulation induced by ZnO NPs was imaged and calculated using the confocal microscopy (36 cells). Scale bar, 10 μm. **c** Time course analysis of phosphorylated and total Bcl-2 protein expression in ZnO NPs-treated PC12 cells. **d** Effects of JNK on ZnO NPs-induced phosphorylation of Bcl-2 and disruption of Bcl-2/Beclin 1 complex. Bcl-2 co-immunoprecipitation with Beclin 1 was detected with a monoclonal anti-Beclin 1 antibody. Input represented the total amount of p-Bcl-2 and p-JNK detected in whole-cell lysates by Western blotting analysis. Images were representative of at least three independent experiments, and the densitometry of the above bolts was shown in Figure S8E-G

JNK has been documented to play an important role in autophagy formation, although the underlying mechanism is unclear [44]. Having identified that the time of JNK activation by ZnO NPs was prior to the conversion of LC3B-I to LC3B-II (Figs. 4e and 5b), and PI staining results indicated that compared with 3-MA, SP600125 reduced the cytotoxicity of ZnO NPs to the same extent (Figs. 4a and 5c). We hypothesized a direct relationship between autophagy and JNK pathway. Thereafter, we asked whether ZnO NPs-induced autophagy required JNK activation. As showed in Fig. 6a, the inhibition of JNK by SP600125 significantly alleviated ZnO NPs-induced autophagy as the markedly decreased level of LC3B-II, indicating a role of JNK in the regulation of autophagy. This phenomenon was further confirmed by GFP-LC3 transfected cells. Cells treated with ZnO NPs for 6 h displayed more GFP-LC3 puncta formation than the control, whereas SP600125 decreased the number of GFP-LC3 puncta induced by ZnO NPs (Fig. 6b). However, the mechanism by which ZnO NPs regulate the interaction between JNK and autophagy remains unknown. The phosphorylation of Bcl-2 at multiple residues (such as T69, S70 and S87) inhibits its binding to BH3 domain-containing protein Beclin 1 and promotes autophagy [45]. We found that the phosphorylation of Bcl-2 at S70 residue occurred after 1 h incubation with ZnO NPs, and the level of total Bcl-2 was not changed (Fig. 6c). This observation led us to wonder whether Bcl-2 phosphorylation is the key to JNK-mediated autophagy. Indeed, the inhibition of JNK by SP600125 significantly inhibited ZnO NPs-induced phosphorylation of Bcl-2, and in reciprocal IP assay, SP600125 incubation almost completely blocked the ZnO NPs-induced disassociation of Beclin 1 from Bcl-2 (Fig. 6d), implying a JNK-dependent negative-regulation of the binding of Bcl-2 to Beclin 1 in ZnO NPs -treated cells. Moreover, SP600125 had no effect on cell uptake of ZnO NPs (Figure S5). Collectively, these results demonstrate that the prolonged JNK activation leads to Bcl-2 phosphorylation, the dissociation of Beclin 1 from Bcl-2, which mediates the autophagosome formation during ZnO NPs exposure.

### The impaired autophagosomal-lysosomal fusion implicates the blockade of autophagic flux by ZnO NPs

**Fig. 7:**
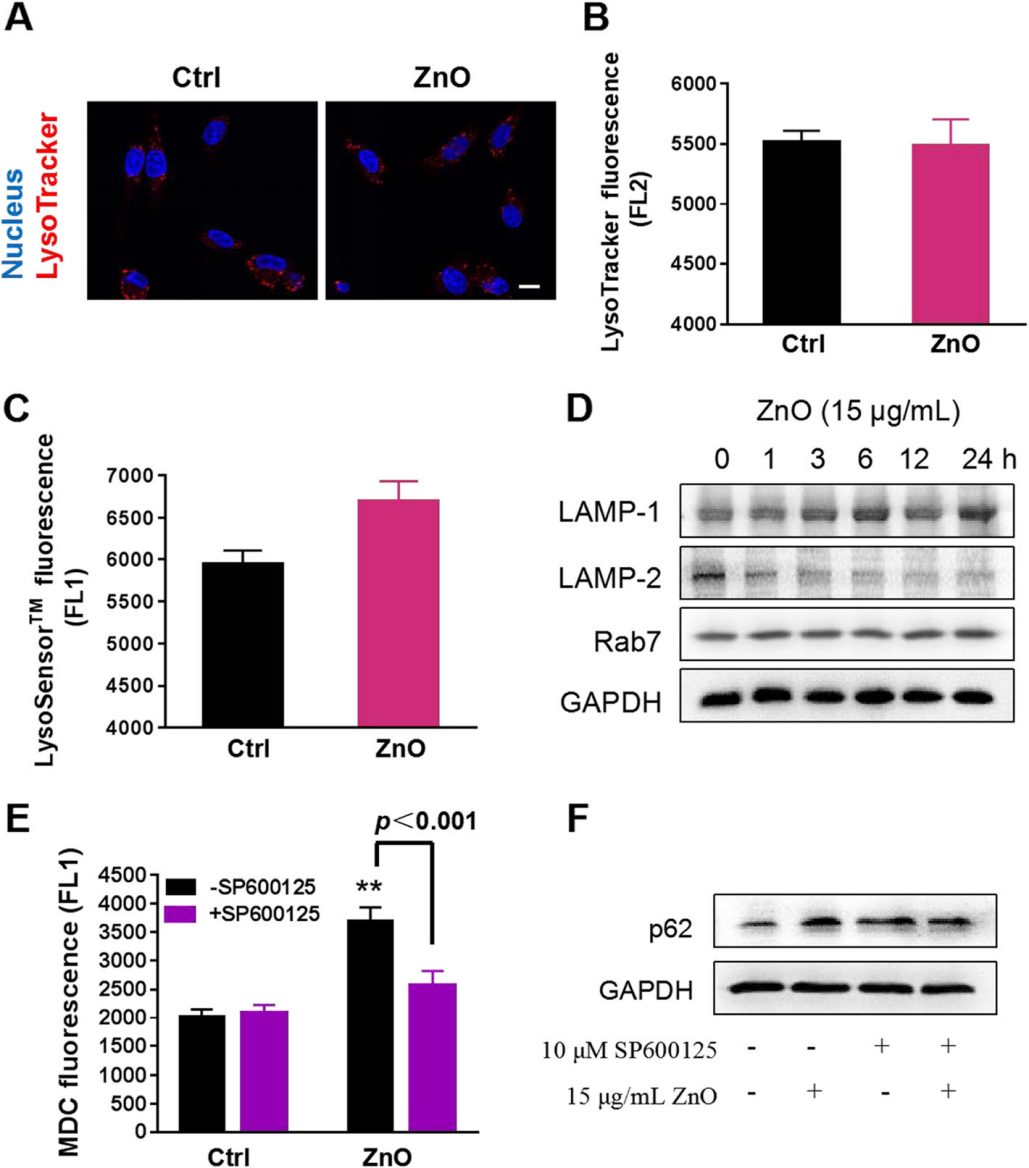
Aberrant LAMP-2 expression results in impaired autophagic flux. **a** Confocal microscopic fluorescence images of lysosomal characters by LysoTracker Red DND-99 staining. PC12 cells were treated with 15 μg/mL ZnO NPs for 12 h, and 32 cells were analyzed to select the representative images. Scale bar, 10 μm. Flow cytometry analysis of lysosomes **(b)** and lysosomal pH **(c)** in ZnO NPs-treated PC12 cells. The cells were treated as above, then stained by LysoTracker Red DND-99 and LysoSensor Green DND 189, respectively. **d** Effects of ZnO NPs on the lysosomal-related protein expression of LAMP-1, LAMP-2 and Rab7. Cell lysates were analyzed by Western blotting. **e** Flow cytometry analysis of autophagosomes by MDC staining in ZnO NPs-induced PC12 cells for 6 h with or without 10 μM SP600125 pre-treatment for 1 h. Data from at least three independent experiments were expressed as the means ± SD. ***p < 0.001 compared with the untreated control. **f** Effect of the JNK inhibitor SP600125 on the level of p62. Cells were treated with 15 μg/mL ZnO NPs for 6 h in the presence or absence of SP600125 pre-treatment for 1 h. Western blotting was performed to determine the level of p62 expression. The densitometry of the above bolts from at least three independent experiments was shown in Figure S9A and B

The above observation has clarified the mechanism of ZnO NPs-induced autophagy initiation. However, ZnO NPs may independently target multiple steps in the autophagy pathway and still result in an overall disruption of autophagy process. Therefore, we continued to explore the mechanism of ZnO NPs-mediated impairment of autophagic flux. The reduced lysosomal function and autophagosomal-lysosomal fusion disorders are upstream triggers in the blockade of autophagic flux [46, 47]. To test these alternatives, we first determined whether ZnO NPs interfered with lysosomal function by using lysosomal probes to examine the amount and acidification of lysosomes. Confocal microscopy images showed no significant changes in the number and size of lysosomes in PC12 cells after exposure to ZnO NPs (Fig. 7a). The same effect could be shown by measuring the fluorescence intensity by flow cytometry (Fig. 7b). Since the degradation capacity of lysosomes has been reported to be dependent on its acidic environment [48], cells were stained with LysoSensor Green DND-189 and acridine orange (AO) to measure lysosomal pH. However, flow cytometry data suggested that ZnO NPs exposure has no influence on lysosomal alkalization in PC12 cells (Fig. 7c and S6A). Time-course experiments showed the expression of mature cathepsin D, a lysosomal hydrolase, had no significant change in PC12 cells (Figure S6B). Therefore, the blockade of autophagic flux is independent of impaired lysosomal function.

The process of autophagosomes guided to fuse with lysosomes is required for a successful autophagic flux, and LAMP and Rab7 work together to facilitate autophagosomal-lysosomal fusion [49]. Western blotting results manifested that LAMP-1 and Rab7 had only slight changes, whereas LAMP-2 expression was significantly reduced in a time-dependent manner upon ZnO NPs exposure (Fig. 7d), suggesting the impairment in the fusion of autophagosomes to lysosomes, which is in agreement with the previous study [15]. In conclusion, ZnO NPs do not affect lysosomal function, but the inhibition of autophagosomal-lysosomal fusion may prevent autophagy clearance and impair autophagic flux in PC12 cells. Interestingly, when SP600125 inhibited ZnO NPs-induced autophagosome accumulation (Fig. 7e and S7), by monitoring the level of p62, ZnO NPs-induced blockade of autophagic flux was improved in the presence of JNK inhibitor SP600125 (Fig. 7f). As compared with the control group, the p62 level in the SP600125 group was not decreased, it can be excluded that SP600125 directly reduced the protein expression of p62 (Fig. 7f). Combined with the results in Fig. 6, we conclude that JNK activation not only induces the formation of autophagosomes, but also impairs the autophagic flux, and the mechanism needs to be further explored.

### ZnO NPs regulate autophagic flux through the dissolution of zinc ions and JNK-autophagy positive feedback loop

**Fig. 8:**
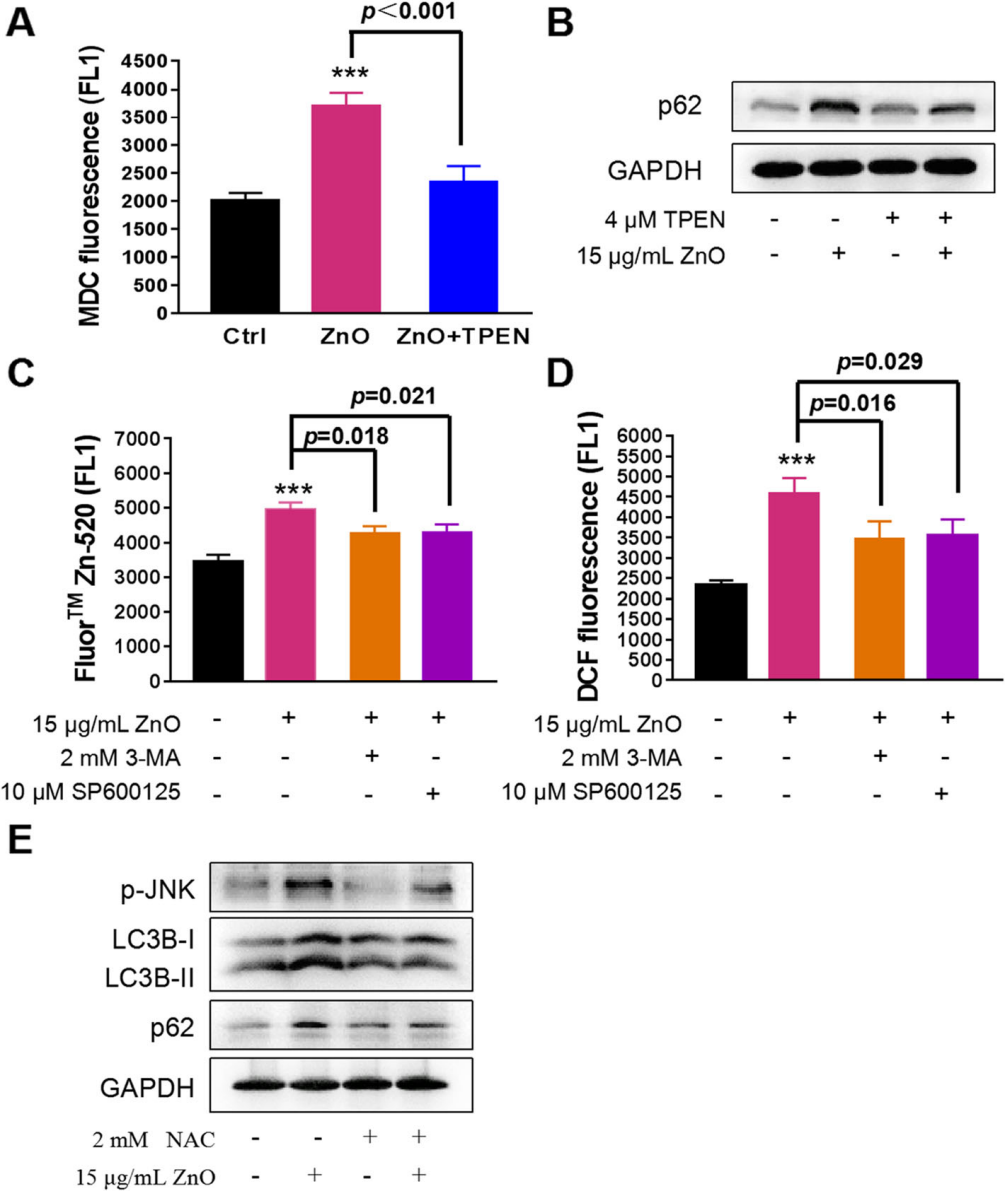
ZnO NPs regulates autophagic flux in an iron and ROS-dependent manner. Flow cytometry analysis of autophagosomes (MDC staining) (**a**) and Western blotting analysis of p62 expression level (**b**) in ZnO NPs-induced PC12 cells for 6 h with or without TPEN pre-treatment for 30 min. Effects of 3-MA and SP600125 on intracellular zinc ions detected by zinc ions indicator Fluor™ Zn-520, AM staining **(c)** and ROS level detected by DCFH-DA staining (**d**) in ZnO NPs-treated PC12 cells. Cells were pretreated with 3-MA or SP600125 for 1 h, followed by exposure to 15 μg/mL ZnO NPs for 6 h. Data from at least three independent experiments were expressed as the means ± SD. ***p < 0.001 compared with the untreated control. **e** The inhibitory effects of NAC on the expression of p-JNK, LC3B conversion and p62 were determined by Western blotting. Cells were treated with 15 μg/mL ZnO NPs for 6 h in the presence or absence of NAC pre-treatment for 1 h. The densitometry of the above bolts from at least three independent experiments was shown in Figure S9C and D

**Fig. 9:**
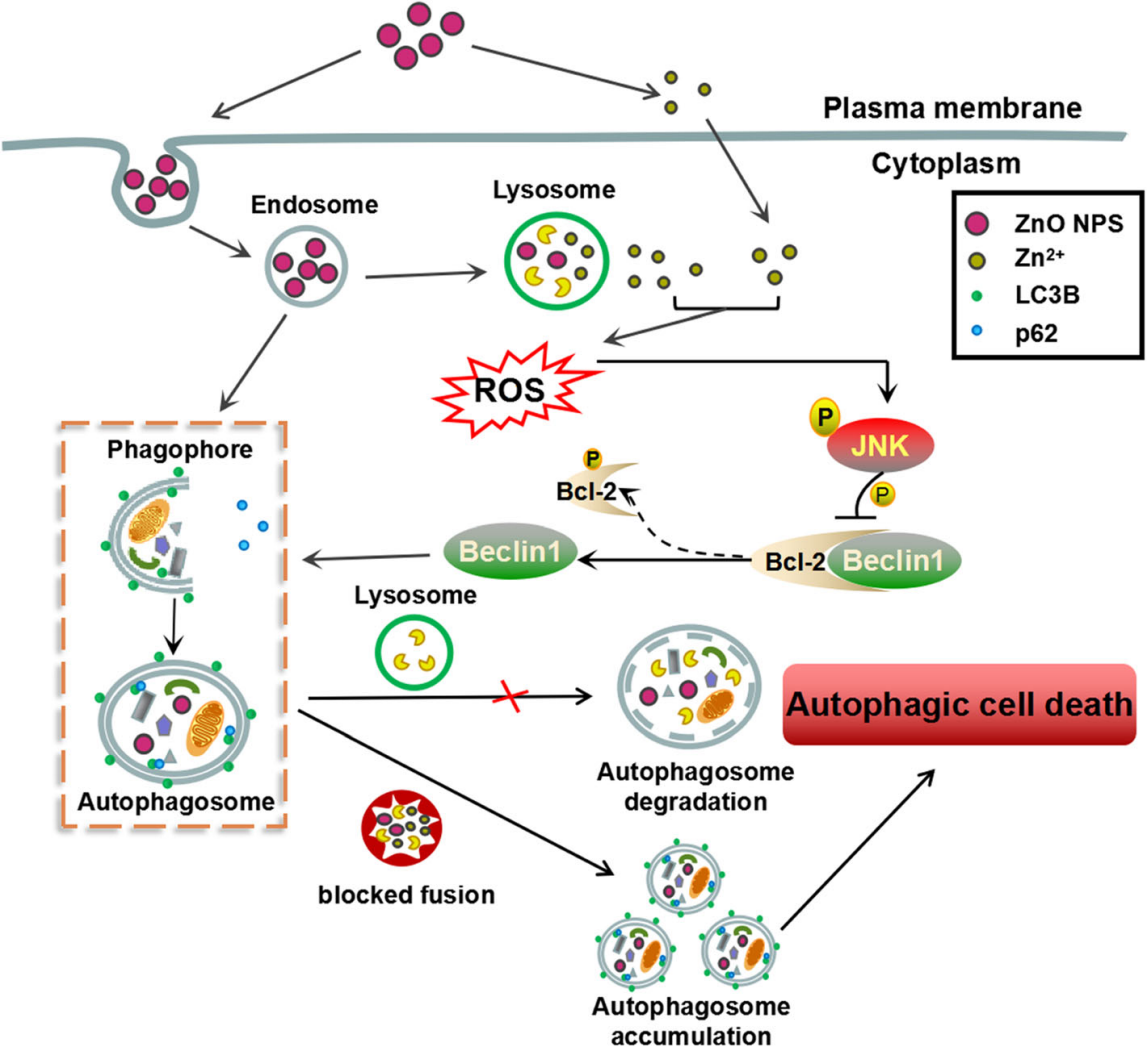
Schematic of the proposed mechanisms of ZnO NPs-inducing autophagic cell death in PC12 cells. ZnO NPs are delivered into lysosomes, and subsequently dissolved in lysosomes to release zinc ions. Zinc ions (intracellular or extracellular) is the crucial factor triggering oxidative stress. Furthermore, the JNK is activated in response to cellular ROS, and JNK activation promotes the initiation of autophagy by phosphorylating Bcl-2, leading to the release of Beclin 1 from Bcl-2. Autophagy is a process of lysosomal-mediated cellular self-digestion. In addition, when the expression of autophagosomal-lysosomal fusion related protein LAMP-2 was affected by excessive zinc ions, autophagic flux will be significantly impaired, aggravating the accumulation of autophagosomes and then inducing autophagic stress. Furthermore, autophagy is also involved in transporting ZnO NPs into lysosomes, accelerating ROS production, enhancing JNK activation, and finally increasing autophagy to form a positive feedback mechanism that contributes to ZnO NPs-induced autophagic cell death

Although zinc is a necessary element, the excessive accumulation of local zinc ions causes lysosome damage [50]. Our observations showed that intracellular zinc ions concentrated specifically in lysosomes (Fig. 1e), implicating the lysosomal vulnerability upon ZnO NPs exposure. Considering the results in Fig. 7, we proposed that the dynamic regulation of autophagic flux may be related to zinc ions shed by ZnO NPs. To address whether zinc ions play an essential role in ZnO NPs-induced autophagic flux impairment, we examined the effects of TPEN on autophagosome accumulation and autophagosome removal. MDC staining showed the chelating of intracellular zinc ions by TPEN effectively decreased the level of autophagosomes (Fig. 8a) and enhanced the degradation of p62 (Fig. 8b), suggesting that zinc ions participated in ZnO NPs-induced autophagic flux impairment. To better understand the interplay between zinc ions and autophagic flux, we in turn detected the levels of zinc ions after autophagy inhibition. 3-MA or SP600125 reduced the release of zinc ions (Fig. 8c) and then mitigated ZnO NPs-induced excessive ROS (Fig. 8d), indicating that autophagy facilitated ZnO NPs to be delivered into lysosomes for dissolution. Moreover, the regulation of autophagic flux by ZnO NPs was attributed to the production of ROS. As shown in Fig. 8e, NAC prevented ZnO NPs-induced JNK activation, autophagy initiation and blockade of autophagic flux. These results showed that ZnO NPs-induced autophagic flux impairment involves zinc ions-ROS-JNK-autophagy positive feedback loop in PC12 cells. Collectively, a mechanical toxicological pathway for ZnO NPs in PC12 cells was shown in the schematic of Fig. 9. ZnO NPs are incorporated into lysosomes via endosomes to shed zinc ions, resulting in the perturbation of cytosolic zinc homeostasis, ROS generation, JNK activation and autophagy initiation. Moreover, ZnO NPs use autophagy to further release zinc ions and consequently enhance the inhibition of autophagosomal-lysosomal fusion, resulting in the accumulation of autophagosomes and eventually autophagic cell death.

## Conclusion

Our results highlighted the key role of autophagosome accumulation as a critical mechanism in ZnO NPs-induced neurotoxicity. The zinc ions-ROS-JNK-autophagy positive feedback loop and the blockade of autophagosomal-lysosomal fusion induced by ZnO NPs lead to the excessive accumulation of autophagosomes, ultimately resulting in autophagic cell death. Our results indicate that modulating the autophagy process may alleviate the toxicity of ZnO NPs.

## Supplementary information

**Additional file 1: Supplementary Figure 1.** Characterization of ZnO NPs. **Supplementary Figure 2.** The cytotoxicity and intracellular zinc ion levels of ZnO NPs and ZnCl_2_ in PC12 cells. **Supplementary Figure 3.** Effects of ZnO NPs on autophagy-related signaling pathway. **Supplementary Figure 4.** Effect of ZnO NPs on the activation of c-Jun. **Supplementary Figure 5.** Effect of 3-MA and SP600125 on the uptake of ZnO NPs. **Supplementary Figure 6.** Effects of ZnO NPs on lysosomal pH and cathepsin B maturation. **Supplementary Figure 7.** Representative confocal microscopic fluorescence images showing the effect of the JNK inhibitor on autophagosome formation. **Supplementary Figure 8.** The densitometry analysis of Western blotting. **Supplementary Figure 9.** The densitometry analysis of Western blotting. **Supplementary Table 1.** Summary of the physical properties of ZnO NPs

## Data Availability

All data analyzed within this study are included either in the manuscript or in the additional supplementary files.
