# Supplementary material for: Zinc oxide nanoparticles effectively regulate autophagic cell death by activating autophagosome formation and interfering with their maturation
Source: Part Fibre Toxicol. 2020 Sep 18;17:46. doi: 10.1186/s12989-020-00379-7 (PMC7501661; doi:10.1186/s12989-020-00379-7)

**SUPPORTING INFORMATION**

**Zinc Oxide Nanoparticles Effectively Regulate Autophagic Cell Death by Activating Autophagosome Formation and Interfering with Their Maturation**

Zixuan Liu, Xuying Lv, Lei Xu, Xuting Liu, Xiangyu Zhu, Erqun Song, Yang Song*

Key Laboratory of Luminescence Analysis and Molecular Sensing (Southwest University), Ministry of Education, College of Pharmaceutical Sciences, Southwest University, Chongqing, People’s Republic of China, 400715

*Corresponding Author: College of Pharmaceutical Sciences, Southwest University, Beibei, Chongqing, People’s Republic of China, 400715. Tel: +86-23-68250371. Fax: +86-23-68251225. E-mail: songyangwenrong@hotmail.com

**Table of Contents:**

**Materials and methods**.……………………………...............................................….…...Page 3-4

**Supplementary Figure 1.** Characterization of ZnO NPs.……………………………........Page 5

**Supplementary Figure 2.** The cytotoxicity and intracellular zinc ion levels of ZnO NPs and ZnCl_2_ in PC12 cells.………………………………………………...………………………….…......Page 6

**Supplementary Figure 3.** Effects of ZnO NPs on autophagy-related signaling pathway...Page 7

**Supplementary Figure 4.** Effect of ZnO NPs on the activation of c-Jun………………....Page 8

**Supplementary Figure 5.** Effect of 3-MA and SP600125 on the uptake of ZnO NPs…....Page 9

**Supplementary Figure 6.** Effects of ZnO NPs on lysosomal pH and cathepsin B maturation.Page 10

**Supplementary Figure 7.** Representative confocal microscopic fluorescence images showing the effect of the JNK inhibitor on autophagosome formation.......................... ............................Page 11

**Supplementary Figure 8.** The densitometry analysis of Western blotting. ............................. Page 12

**Supplementary Figure 9.** The densitometry analysis of Western blotting. ............................. Page 13

**Supplementary Table 1.** Summary of the physical properties of ZnO NPs..…………….....Page 14

**Materials and methods**

**Reagents**

Acridine orange (AO) and monodansylcadaverine (MDC) were supplied by Sigma-Aldrich (Missouri, USA). 3-Methyladenine (3-MA) and SP600125 were obtained from Selleckchem (Texas, USA). Hoechst 33258 was purchased from Solarbio (Beijing, China). Antibody to cathepsin D was obtained from Santa Cruz Biotechnology (Santa Cruz, CA). Antibody to c-Jun was supplied by Bimake (Texas, USA). Antibody to phosphorylated AMP-activated protein kinase (p-AMPK, Thr172) was supplied by Biosynthesis Biotechnology (Beijing, China). Antibody to phosphorylated mammalian target of rapamycin (p-mTOR, Ser2448) was purchased from Ruiying Biological (Suzhou, China).

**Characterization of** **ZnO NPs**

Zeta potential and hydrodynamic size of ZnO NPs in suspension were measured by dynamic light scattering (DLS) technique using a ZEN3700 instrument (Malvern Instruments, UK).

**Zinc ions release assay in buffering system**

For zinc ions concentrations in the supernatant of ZnO NPs suspension detection, ZnO NPs were dissolved in water or complete cell culture medium at a concentration of 15 μg/mL, and incubated at 37°C for the indicated times. Then, the suspension was centrifuged at 18, 000 rpm for 1 h. Finally, the supernatant was collected for detection. The concentration of zinc ions was determined using atomic absorption spectroscopy (AAS) in the graphite furnace mode (TAS-990, Persee, China). Each experiment was performed in triplicate.

**AO staining**

After treatment of ZnO NPs, cells were stained by AO (0.1 µg/mL) for 30 min. Then cells were resuspended and fluorescence was determined with a BD FACS Melody^TM^ flow cytometer.

**Visualization of MDC-labeled vacuoles**

Autophagosomes were detected with MDC staining. Briefly, after treatment of ZnO NPs, cells were collected and incubated with 50 μM MDC for 30 min. Then cells were washed twice with PBS, followed by incubation with 1 mg/mL Hoechst 33258 for 20 min in the dark. MDC fluorescence was examined using the confocal microscope (Nikon, Japan) with an excitation wavelength of 405 nm for nucleus and 488 nm for autophagosomes.

**Statistical analysis**

All data, generated by at least three independent experiments, was presented as mean ± standard deviations (SD). The statistical significance of the differences was analyzed in GraphPad Prism 7.0 software by one-way ANOVA followed by Tukey's multiple comparisons test, and *p* values less than 0.05 was considered statistically significant.

**Supplementary figure legends**

**Supplementary Figure 1. Characterization of ZnO NPs.** **(A)** ZnO NPs morphology and size were detected using TEM. Scale bar, 200 nm. **(B)** DLS was used to determine the size distribution of ZnO NPs in water and complete cell culture medium. **(C)** Time course of ZnO NPs dissolution in water and complete cell culture medium at 37°C. Zinc ions concentrations in supernatants of 15 μg/mL ZnO NPs suspensions within the 1 h time frame were detected by AAS.


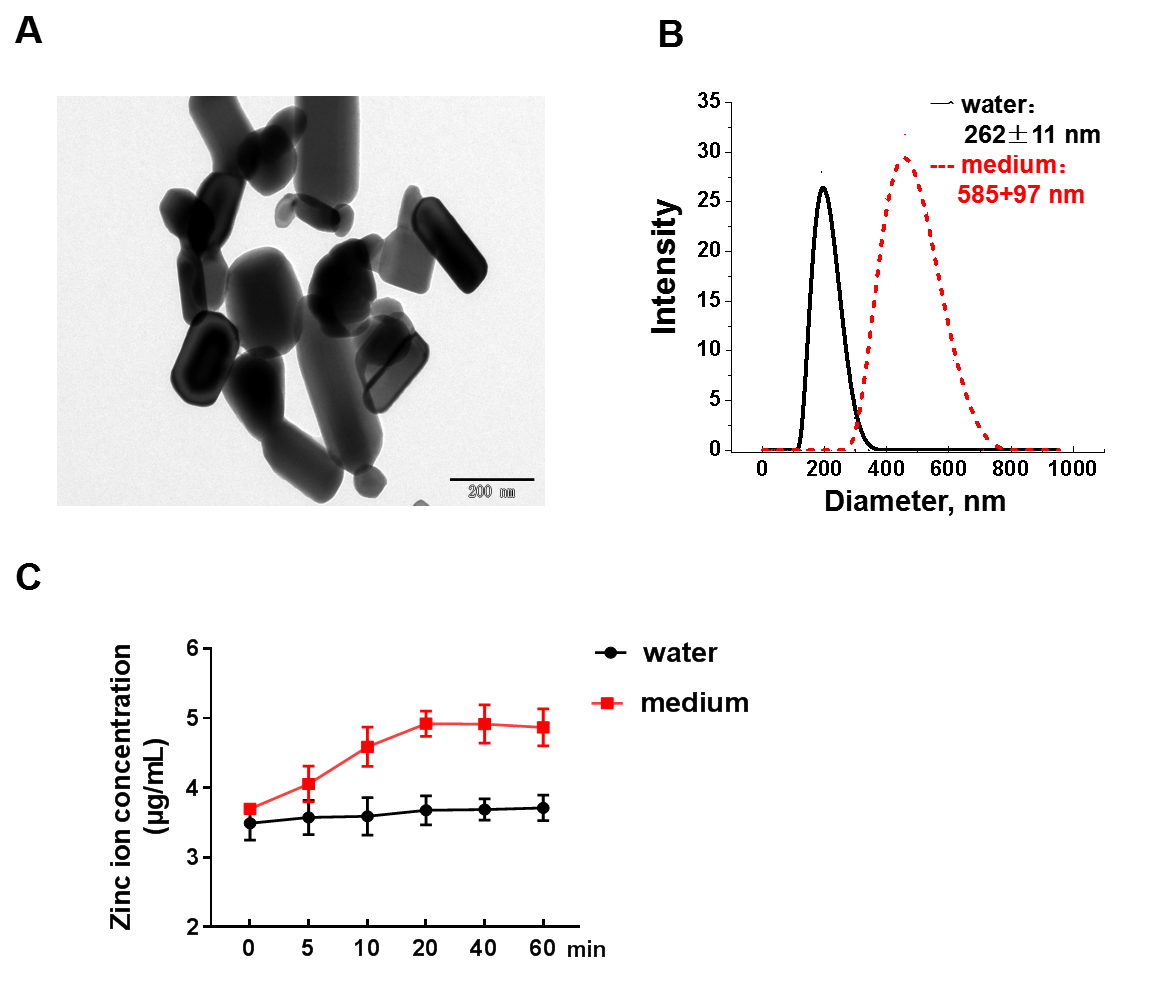


**Supplementary Figure 2. The cytotoxicity and intracellular zinc ion levels of ZnO NPs and ZnCl_2_ in PC12 cells.** **(A)** CCK8 analysis of PC12 cells treated with the indicated doses of ZnO NPs or ZnCl_2_. Cell viability was determined at 24 h after ZnO NPs or ZnCl_2_ treatment. **(B)** The available intracellular zinc ions with increasing ZnO NPs or ZnCl_2_ concentrations were measured by AAS quantification. **(C)** Intercorrelation of cell viability and intracellular free zinc ions level after treatment with ZnCl_2_ for 24 h. Data from at least three independent experiments were expressed as the means± SD.


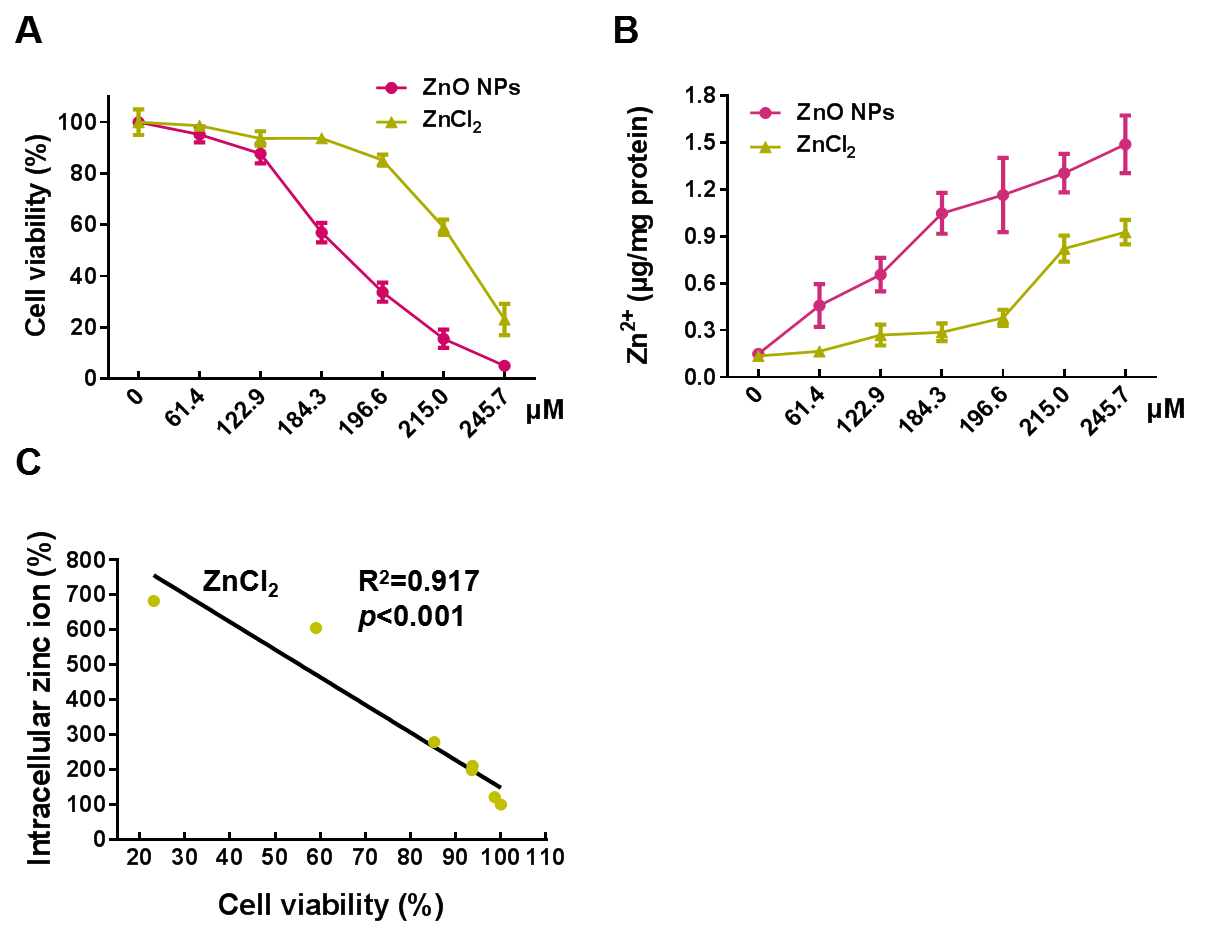


**Supplementary Figure 3**. **Effects of ZnO NPs on autophagy-related signaling pathway.** Expression of p-mTOR **(A)** and p-AMPK **(B)** were detected by Western blotting. Densitometry results of the blots for p-mTOR (S2448) and p-AMPK (T172) from at least three independent experiments were calculated using ImageJ software. **p*<0.05, ***p*<0.01, ****p*<0.001 compared with the untreated control.


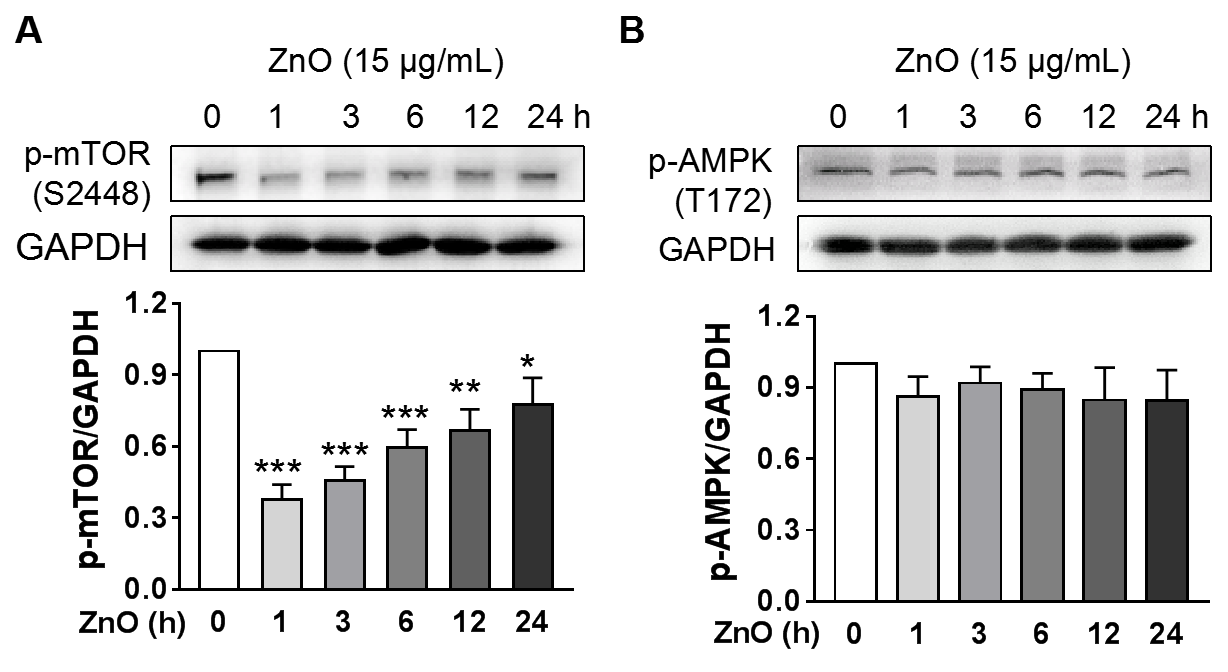


**Supplementary Figure 4.** **Effect of ZnO NPs on the activation of c-Jun.** PC12 cells were treated with 15 μg/mL ZnO NPs for the indicated times, and the activation of c-Jun was detected by Western blotting. Densitometry result of the blot for p-c-jun from at least three independent experiments was calculated using ImageJ software. ****p*<0.001 compared with the untreated control.


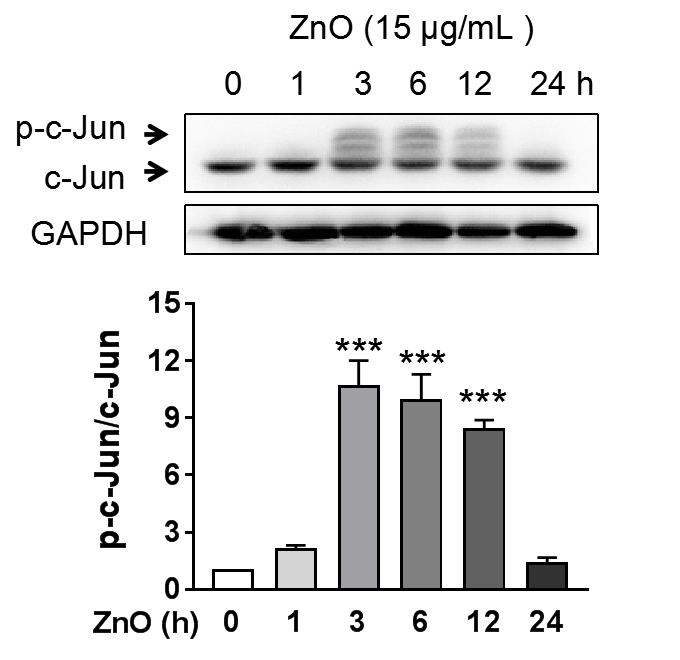


**Supplementary Figure 5. Effect of 3-MA and SP600125 on the uptake of ZnO NPs.** ZnO NPs-induced PC12 cells for 2 h with or without 2 mM 3-MA or 10 μM SP600125 pre-treatment for 1 h. SSC quantified the uptake of ZnO NPs, and the data were expressed as subtracting the corresponding control group.


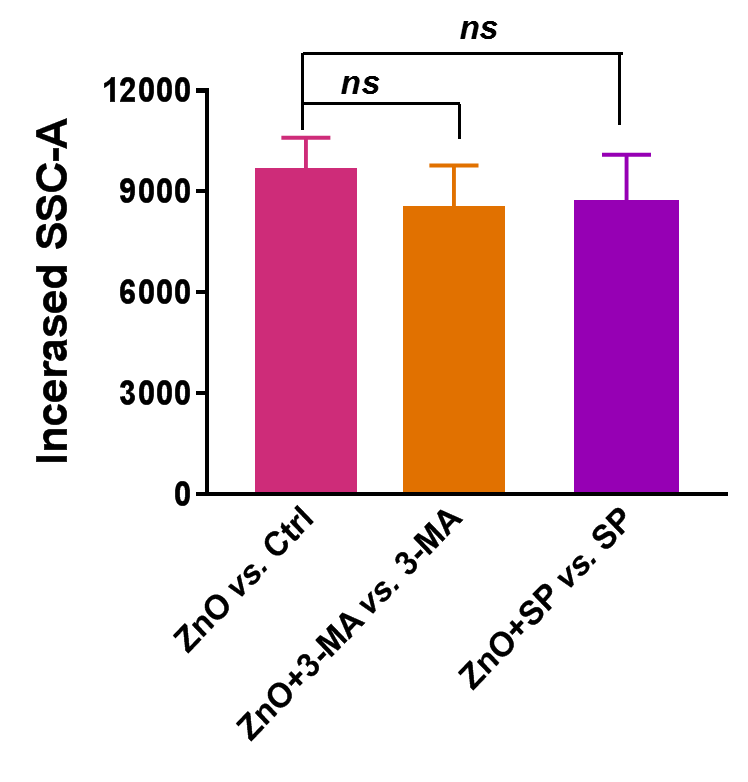


**Supplementary Figure 6.** **Effects of ZnO NPs on lysosomal pH and cathepsin B maturation. (A)** After treatment with ZnO NPs for 12 h, cells were subjected to pH-dependent fluorescent dye acridine orange (AO) to detect lysosomal acidification. Data from at least three independent experiments were expressed as the means ± SD. **(B)** Effect of ZnO NPs on the maturation of cathepsin B. Densitometry results of the blot for Cathepsin D were calculated using ImageJ software.


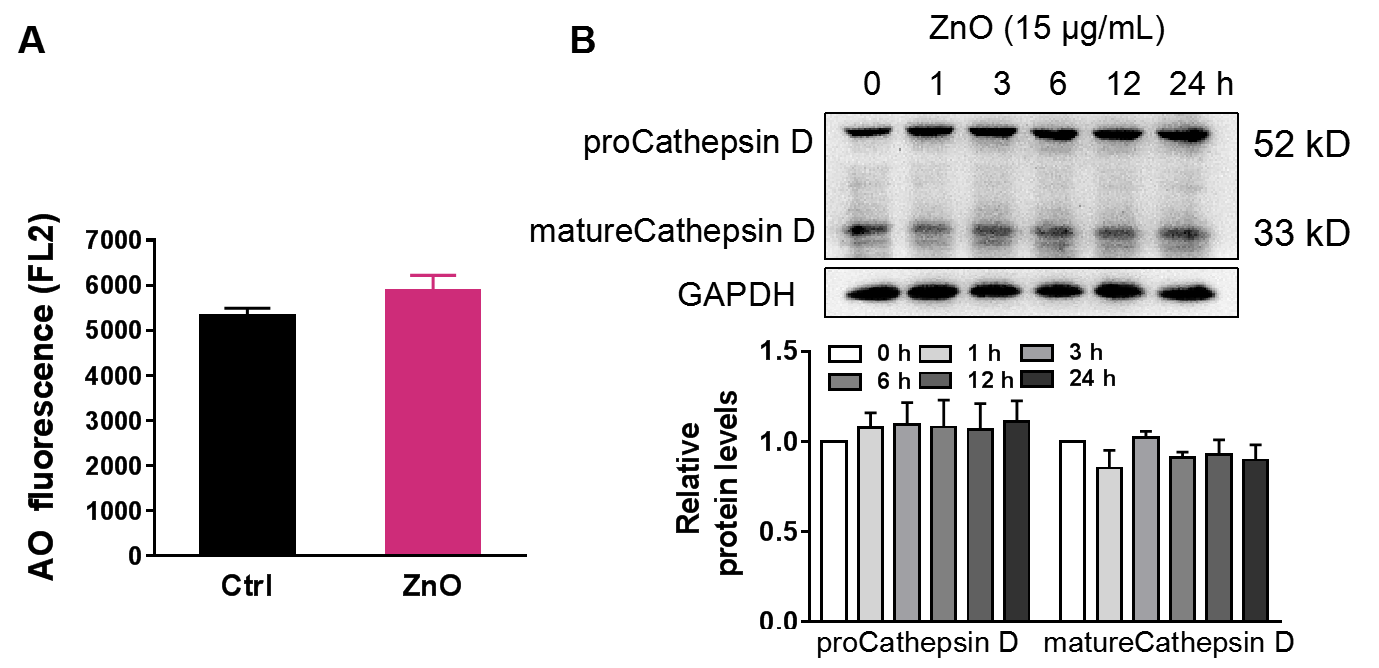


**Supplementary Figure 7. Representative confocal microscopic fluorescence images showing the effect of the JNK inhibitor on autophagosome formation (MDC staining)**. Cells were treated with 15 μg/mL ZnO NPs for 6 h in the presence or absence of 10 μM SP600125 pre-treatment for 1 h, and 45 cells were analyzed to select the representative images. Scale bar, 10 μm.


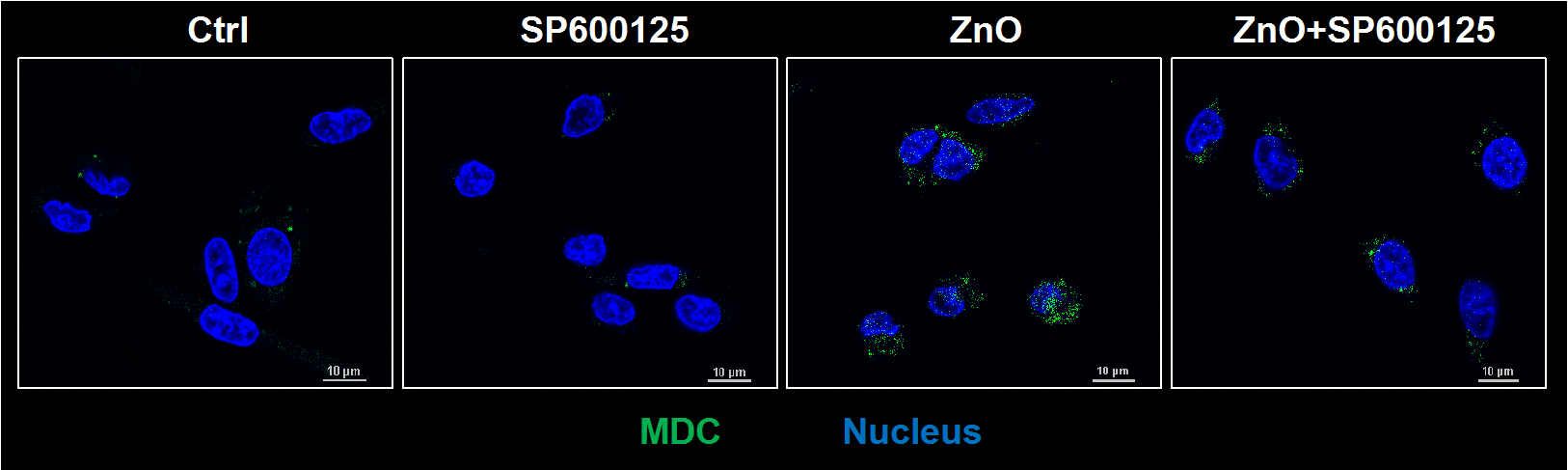


**Supplementary Figure 8. The densitometry analysis of Western blotting.** **p*<0.05, ***p*<0.01, ****p*<0.001 compared with the untreated control. *ns*, not significant.


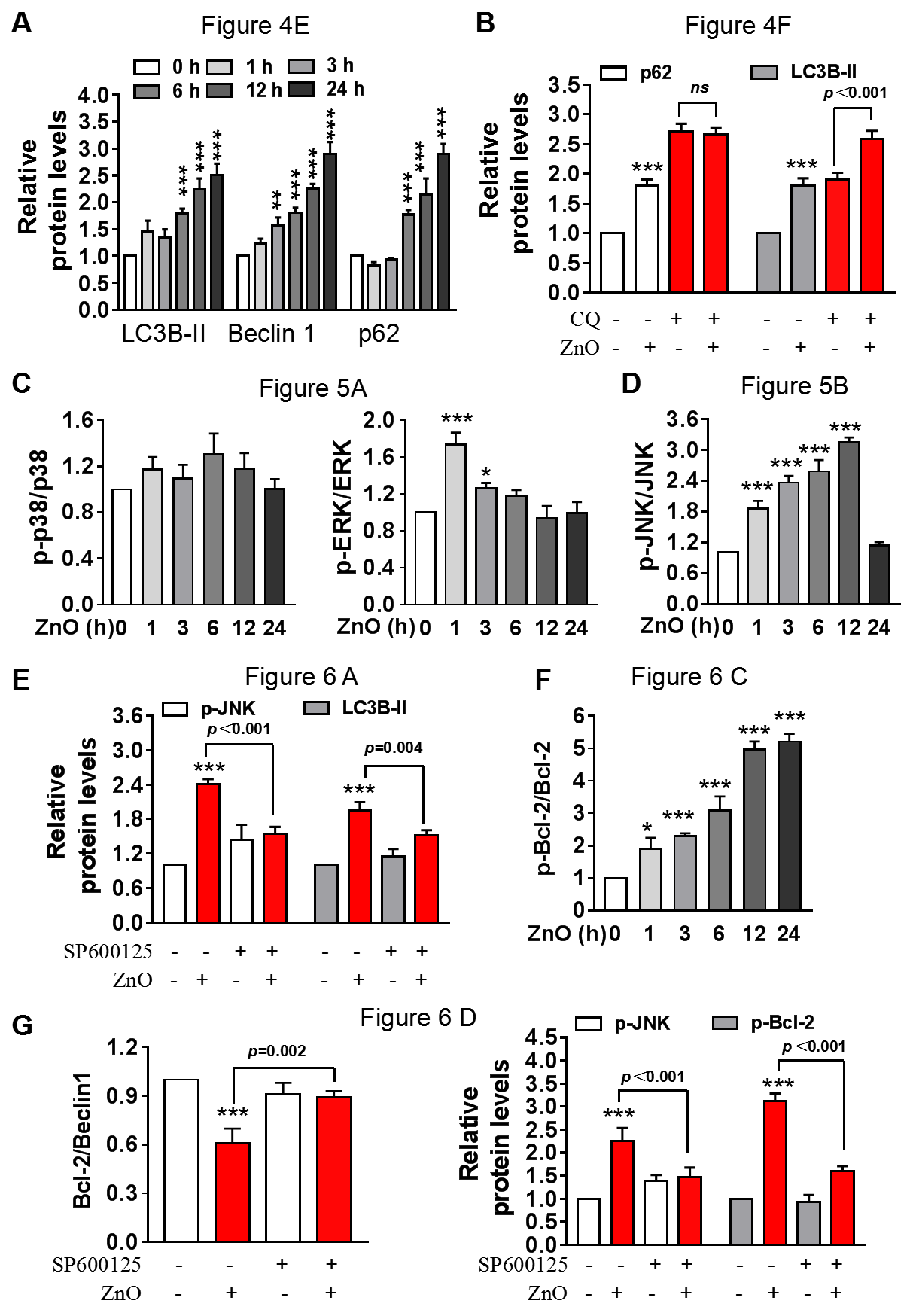


**Supplementary Figure 9. The densitometry analysis of Western blotting.** Densitometry results of the blots from at least three independent experiments were calculated using ImageJ software. ****p*<0.001 compared with the untreated control.


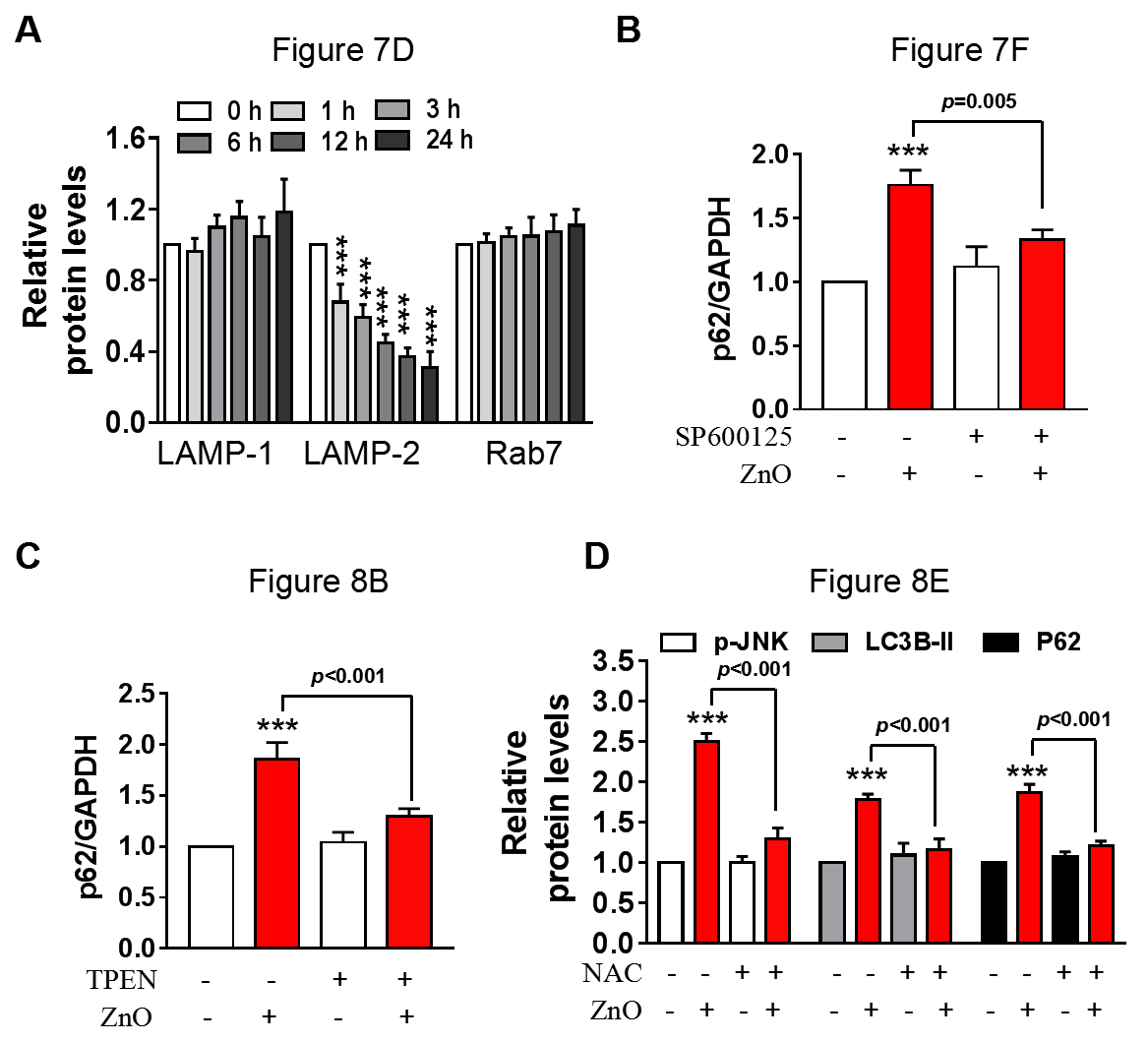


### Supplementary Table 1. Summary of the physical properties of ZnO NPs.


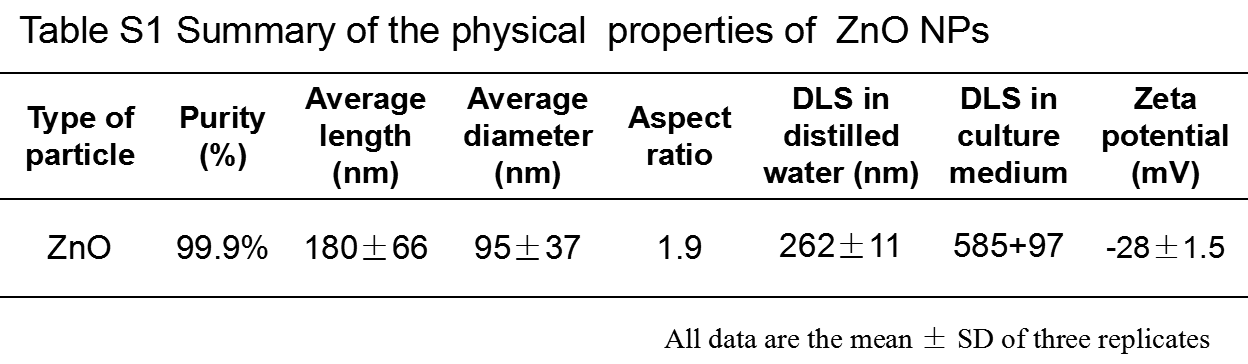

Supplement: Supplementary file 1 — Additional file 1: Supplementary Figure 1. Characterization of ZnO NPs. Supplementary Figure 2. The cytotoxicity and intracellular zinc ion levels of ZnO NPs and ZnCl2 in PC12 cells. Supplementary Figure 3. Effects of ZnO NPs on autophagy-related signaling pathway. Supplementary Figure 4. Effect of ZnO NPs on the activation of c-Jun. Supplementary Figure 5. Effect of 3-MA and SP600125 on the uptake of ZnO NPs. Supplementary Figure 6. Effects of ZnO NPs on lysosomal pH and cathepsin B maturation. Supplementary Figure 7. Representative confocal microscopic fluorescence images showing the effect of the JNK inhibitor on autophagosome formation. Supplementary Figure 8. The densitometry analysis of Western blotting. Supplementary Figure 9. The densitometry analysis of Western blotting. Supplementary Table 1. Summary of the physical properties of ZnO NPs [file 12989_2020_379_MOESM1_ESM.doc]
